# Fractionating proteins with nitrite-reducing activity in “*Candidatus* Kuenenia stuttgartiensis” strain CSTR1

**DOI:** 10.3389/fmicb.2025.1483703

**Published:** 2025-02-26

**Authors:** Emea Okorafor Ude, Pranathi Sure, Rimjhim Rimjhim, Lorenz Adrian, Chang Ding

**Affiliations:** ^1^Department of Molecular Environmental Biotechnology, Helmholtz Centre for Environmental Research – UFZ, Leipzig, Germany; ^2^Chair of Geobiotechnology, Technische Universität Berlin, Berlin, Germany

**Keywords:** shotgun proteomics, fractionation, anammoxosome, planktonic cultivation, nitrogen cycle

## Abstract

The anammox bacteria “*Candidatus* Kuenenia stuttgartiensis” (*Ca.* Kuenenia) are able to gain energy by combining ammonium and nitrite to produce nitrogen gas, which is an ecologically and technically significant activity process. In this reaction, nitric oxide serves as a recognized intermediate in the reduction of nitrite, which is subsequently combined with ammonium to produce hydrazine. However, the enzyme that converts nitrite to nitric oxide remains elusive. In this study, we investigated the nitrite-reducing activity in “*Ca.* Kuenenia stuttgartiensis” strain CSTR1 to identify candidates for such an enzyme. An optimized *in vitro* assay was established to measure nitrite-reducing activities, with which we followed the activity in protein fractions obtained from various fractionation methods. Separation of the cell extract of strain CSTR1 with size exclusion chromatography yielded active fractions corresponding to a molecular size range of 150–200 kDa. Several proteins coeluted with the nitrite-reducing activity, including the hydroxylamine dehydrogenase HOX, an NADP-dependent isopropanol dehydrogenase (Adh), an electron-transfer 4Fe-4S subunit protein (Fcp), and a nitric oxide detoxifying flavorubredoxin (NorVW). However, further separation of the cell extract with anion exchange chromatography, resulted in much lower activity yields, and activities were distributed among several fractions. In addition, fractionation of cell extracts using ultracentrifugation and ultrafiltration linked the activity to HOX, but could not exclude the involvement of other proteins in the activity. Overall, our results suggest that the molecular mechanism for nitrite reduction in “*Ca.* Kuenenia” strains is more complex than that currently described in the literature. Nitrite reduction appears to be strongly associated with HOX but may additionally require the participation of other proteins.

## Introduction

Anaerobic ammonium oxidation (anammox) is an essential microbially-catalyzed process in the global nitrogen cycle, where nitrite and ammonium are transformed into dinitrogen (Kartal et al., 2011; Kuypers et al., 2003). So far, several taxa of anammox bacteria have been identified all of which belong to the phylum Planctomycetota (Cuecas et al., 2024; Lodha et al., 2021; Zhao et al., 2023). In the typical anammox metabolism, first, nitrite is reduced to nitric oxide (NO) or hydroxylamine (NH_2_OH). Then NO or NH_2_OH is combined with ammonium to form hydrazine and finally, hydrazine is oxidized to dinitrogen, releasing four electrons (Akram et al., 2019). Apart from being reduced, another portion of nitrite is oxidized to nitrate, releasing two electrons that probably replenish the electron pool used to reduce nitrite (Hu et al., 2019). Three of the four aforementioned steps have been convincingly associated with one or more abundant proteins, that is, nitrite:nitrate oxidoreductase (NXR) for nitrite oxidation to nitrate (Chicano et al., 2021), hydrazine synthase (HZS) for hydrazine production (Dietl et al., 2015; Oshiki et al., 2016), and hydrazine dehydrogenase (HDH) for hydrazine oxidation (Akram et al., 2019). However, the enzymes responsible for nitrite reduction are still not well understood, as the associated gene is less conserved among different genera of anammox bacteria and is even unidentified in some of the anammox species (Peeters and van Niftrik, 2019).

The reduction of nitrite in anammox bacteria follows one of two major pathways, employing different enzymes and varying numbers of electrons. One-electron nitrite reduction to NO was described for the genera *Candidatus* Kuenenia stuttgartiensis “*Ca.* Kuenenia” (Kartal et al., 2011), “*Ca.* Jettenia” (Ali et al., 2015; Hira et al., 2012), and “*Ca.* Scalindua” (Hira et al., 2012; van de Vossenberg et al., 2013), whereas four-electron nitrite reduction to NH_2_OH was suggested for the genus “*Ca.* Brocadia” (Okubo et al., 2021; Oshiki et al., 2016). The canonical nitrite reductase genes *nirS* or *nirK* are indeed missing in the genomes of “*Ca.* Brocadia sinica” (Oshiki et al., 2015; Speth et al., 2016) and “*Ca.* Brocadia fulgida” (Gori et al., 2011). It is so far not clear which nitrite reductase catalyzes the reduction of nitrite to hydroxylamine in “*Ca.* Brocadia” species, and one of the hydroxylamine oxidoreductases (HAOs) was suspected (Oshiki et al., 2016). In “*Ca.* Scalindua” strains (e.g., “*Ca.* Scalindua profunda and *Ca.* Scalindua rubra”), an abundantly expressed NirS could catalyze nitrite reduction (Speth et al., 2017; van de Vossenberg et al., 2013). In “*Ca.* Jettenia caeni,” a copper-based NirK was suggested to reduce nitrite (Hira et al., 2012). NirK was not detected in the proteome of “*Ca.* Jettenia caeni” strain KSU-1 (Ali et al., 2015). This could be due to the high activity of the enzyme, which only requires a low abundance of proteins. Later, the nitrite-reducing activity of NirK was confirmed experimentally (Hira et al., 2020). Apart from NirK, it was also suggested that other enzymes may be involved in nitrite reduction since a copper chelator could not completely inhibit nitrite reduction in “*Ca.* Jettenia caeni” (Ali et al., 2015).

“*Ca.* Kuenenia stuttgartiensis” is the most extensively studied anammox species so far. It has been confirmed that the one-electron nitrite reduction in “*Ca.* Kuenenia stuttgartiensis” produces NO as the key intermediate (Kartal et al., 2011). The genome of “*Ca.* Kuenenia stuttgartiensis” encodes a canonical cytochrome *cd_1_*-containing nitrite reductase (NirS). However, NirS was either barely expressed (Kartal et al., 2011) or only expressed under stress conditions such as high temperature, high nitrite, or starvation (Ding and Adrian, 2020). The low expression levels of NirS under stress-free conditions suggested that “*Ca.* Kuenenia” strains encode another nitrite-reducing enzyme, possibly encoded by one or more genes in the large HAO-encoding gene pool. Using the gene tags that correspond to the genome of strain CSTR1 (Ding and Adrian, 2020) and KUST (Kartal et al., 2013; Zhao et al., 2022), two suspected examples of such enzymes are encoded by the gene locus KsCSTR_49490 (corresponding to kustc0458) and the gene locus KsCSTR_29630 (corresponding to kuste4574). Ferousi et al. (2021) studied the nitrite-reducing activity of the protein encoded by kustc0458. The protein was purified from the strongly enriched mixed culture, and nitrite-reducing activity was observed (0.1 U (= 1.7 nkat) mg^−1^ protein with methyl viologen as the electron carrier, and 0.53 mU (= 8.7 pkat) mg^−1^ protein with phenazine ethosulfate (PES) as the electron carrier). When using PES as the electron carrier, stoichiometric production of nitric oxide from nitrite was observed.

In another study that investigated the role of the highly abundant hydroxylamine dehydrogenase HOX (encoded by kustc1061, corresponding to KsCSTR_43280) (Maalcke et al., 2014), it was demonstrated that HOX oxidized hydroxylamine to nitric oxide, which was proposed as its physiological function to recycle hydroxylamine leaked from the activity of hydrazine synthase (Dietl et al., 2015). Interestingly, the study by Maalcke et al. also showed that HOX could reduce nitrite, at a specific activity of 0.18 U (=3.0 nkat) mg^−1^ protein with methyl viologen as the electron carrier. This particular activity is much higher than the value reported for enzymes encoded by kustc0458 (Ferousi et al., 2021). Therefore, it cannot be excluded that HOX may also play a role in nitrite reduction in “*Ca.* Kuenenia.” All these studies indicate the possibility that anammox bacteria may have multiple enzymes responsible for nitrite reduction, some of which might be encoded by the HAO genes. This might also partly explain the difficulty in identifying one single protein as the responsible nitrite reductase unambiguously.

As mentioned above, studies on nitrite reductase in “*Ca.* Kuenenia” were only done with purified proteins so far. Therefore, it is unclear whether there are other proteins in “*Ca.* Kuenenia” that also catalyze nitrite reduction, and if there are, which proteins contribute the majority to nitrite reduction. We conducted a comprehensive screening of nitrite-reducing activity in the proteome of a “*Ca.* Kuenenia” strain grown in our laboratory. This strain had previously been established as a dominant planktonic population in a semi-continuous stirred tank reactor and was therefore denominated as “*Ca.* Kuenenia stuttgartiensis” strain CSTR1 (Ding and Adrian, 2020; Ding et al., 2018). The proteome of strain CSTR1 was fractionated by size exclusion chromatography (SEC), anion exchange chromatography (AEC), ultracentrifugation, or ultrafiltration. Nitrite-reducing activity was found and linked to several protein candidates.

## Materials and methods

### Chemicals

For the quantification of gasses using gas chromatography–mass spectrometry (GC–MS), NO, nitrous oxide (N_2_O), N_2_, and O_2_ were used as gas standards. NO was prepared by reacting copper with concentrated nitric acid and collected by water displacement. N_2_O was obtained by releasing the carrier gas from a whipped cream can and collected by water displacement. High-purity N_2_ and O_2_ gasses (99.999%) were used to quantify their percentages (v/v) in the gas bottles of NO and N_2_O using GC–MS (as below), to determine the purity of the NO and N_2_O gas standards. All other chemicals were purchased from Sigma–Aldrich (St. Louis, USA) at ACS reagent grade or better.

### Operation of the anammox reactor and preparation of crude cell extracts

A continuously stirred tank reactor cultivating a mixed culture with >85% relative cell counts of “*Ca.* Kuenenia” strain CSTR1 provided planktonic cells for all experiments in this study (Ding and Adrian, 2020; Ding et al., 2018). Cell counts were determined by epifluorescence microscopy on agarose-coated slides (Adrian et al., 2007) in which “*Ca.* Kuenenia” cells can be differentiated from other cells based on their unique c- or ring-shaped light pattern, caused by the lack of fluorescence from the anammoxosome (Ding et al., 2018). The reactor was fed with two media containing nitrite and ammonium, respectively, leading to a final concentration of 60 mM for each in the influent. Nitrite in the reactor was kept all the time below 1.2 mM. The pumps held the hydraulic retention time between 4 and 6 days. The typical effluent cell density was 2.5 × 10^8^ cells ml^−1^. The reactor effluent was stored anoxically at 4°C until usage. Cells were harvested by centrifuging the harvested reactor effluent at 5,000 g for 5 min at 10°C. The obtained cell pellet was washed once using 1× phosphate buffer saline (PBS, pH 7.4, containing 137-mM NaCl, 2.7-mM KCl, 10-mM Na_2_HPO_4_, and 1.8-mM KH_2_PO_4_), and resuspended in the same buffer to achieve 100× cell density (2.5 × 10^10^ cells ml^−1^). The concentrated cells were disrupted by one passage of French press at a pressure of 137 MPa. The effectiveness of disruption was monitored by staining the disrupted cells with SYBR-Green and viewing them on agarose-coated slides with an epifluorescence microscope (Marco-Urrea et al., 2011). Then the disrupted cells were centrifuged at 16,100 g for 10 min at 10°C to obtain the crude cell extract. Pellets containing undisrupted cells were suspended in 1× PBS, and then disrupted again using French press. Cell extracts of both disruption processes were combined and stored as 1-ml aliquots at −80°C until usage. The typical protein concentration of the cell extract from 100× concentrated cells (2.5 × 10^10^ cells ml^−1^) was 2.0 mg ml^−1^ as quantified by the BCA protein assay kit (Pierce, Thermo Fisher Scientific, Massachusetts, USA).

### Fast protein liquid chromatography

Ammonium bicarbonate buffer was used instead of the commonly used phosphate buffer in FPLC runs for compatibility with mass spectrometry detection. Ammonium bicarbonate buffer was always prepared fresh before usage due to the gradual increase in pH after preparation.

For size exclusion chromatography (SEC), a Superdex 200 10/300 GL column (24-ml bed volume, GE Healthcare, USA) was equilibrated with 100-mM ammonium bicarbonate (pH 7.9) until conductivity was stable at 9.3 mS cm^−1^. After equilibrium, 200 μL of whole-cell extracts or fractionated cell extracts were loaded onto the column and separated at a flow rate of 0.2 mL min^−1^.

For anion exchange chromatography (AEC), a Capto Q ImpRes column (1-ml bed volume, Cytiva, Massachusetts, USA) was equilibrated with a starting buffer of 50-mM ammonium bicarbonate (pH 7.9) until conductivity was stable at 4.6 mS cm^−1^. After equilibrium, 500 μL of whole-cell extracts were loaded onto the column and separated at a flow rate of 0.4 mL min^−1^. A linear gradient of 20 mL was used where the elution buffer (50-mM ammonium bicarbonate, 1 M NaCl) increased from 0 to 100%.

All FPLC runs were performed at room temperature. FPLC fractions of 0.1–0.5 mL in volume were collected and stored aerobically on ice before nitrite-reducing activity and protein composition were assessed.

### Ultracentrifugation

Fractionation of whole-cell extracts was carried out using ultracentrifugation on a Beckman Coulter Optima MAX-XP centrifuge (California, USA) equipped with a swing-bucket rotor (MLS-50). The starting volume of the cell extracts was 675 μL. Centrifugation was done at 130,000 g for 3 h at 4°C. After centrifugation, 590 μL of the yellowish supernatant was carefully pipetted out as the cytosolic fraction. The remaining dark red liquid (50 μL) on top of the pellet was then pipetted out and mixed with 250 μL of 1× PBS as the intermediate fraction. The red pellet was resuspended in 600 μL of 1% (w/v) n-dodecyl-β-D-maltoside in 1× PBS and incubated at 4°C for 1 h as the membrane fraction. Cell extracts and all fractions were placed on ice before nitrite-reducing activity and protein composition were assessed.

### Ultrafiltration

The intermediate fraction after ultracentrifugation was further separated by ultrafiltration using centrifugal molecular filters with cutoff sizes of 100 and 3 kDa and processing volume of 0.5 mL (Amicon Ultra, Merck Millipore, USA). Centrifugation was done at 14,000 g for 10 min. Separation was done sequentially first with the 100 kDa filter and then with the 3 kDa filter. The retained fractions in each filter were reconstituted to the original sample volume using 1× PBS. All fractions were evaluated for nitrite-reducing activity, and the fraction that was retained by the 100 kDa filter underwent further separation by SEC.

### *In vitro* nitrite-reducing activity assay

*In vitro* activity assays were performed to quantify nitrite-reducing activities in cells, cell extracts, and fractions obtained after ultracentrifugation, ultrafiltration, or FPLC runs. All steps below (except gas flushing) were performed in an anaerobic tent. The assay conditions for the activity were continuously optimized throughout this study, resulting in slightly different conditions in some experiments. The experimental conditions for each specific activity assay are detailed in the corresponding figure legend. In general, the reaction mixture contained buffer (phosphate or MOPS), ascorbic acid (0.5 or 1 mM) as the reducing agent, 0.2-mM PES or phenazine methosulfate (PMS) as the electron mediator, 300–500-μM ^15^N-labeled or non-labeled sodium nitrite as the substrate, and catalyst (concentrated cells, cell extract, or fractions). PES or PMS was used due to their suitable redox potential that leads to the production of nitric oxide from the reduction of nitrite (Ferousi et al., 2021; Hochstein and Tomlinson, 1988). Ascorbic acid was used as the reductant for the system due to mild reduction behavior and absence of abiotic activity under suitable pH. Phosphate or MOPS was chosen as a buffer due to its appropriate buffering range for the selected pH. All reagents were prepared using anoxic water in the tent. Reactions were prepared in a volume of 300 μL in 20-ml vials, except for the mass balance experiment where 1-ml reactions were prepared in 10-ml vials. Nitrite was added to start the reaction and vials were sealed with butyl rubber stoppers. When ^15^N-labeled nitrite was used, the vials were flushed with helium for 30 s immediately after crimping the vials to remove ^28^N_2_. The headspace pressure was then adjusted to atmospheric pressure by quickly piercing the septum with a 25G needle. Incubation was done without shaking at 30°C in the dark. At the end of incubation, nitrite, ammonium, and/or the headspace composition were determined. The nitrite-reducing activity was gauged either by the total amount of gaseous nitrogen compounds generated (in assays with ^15^N-labeled nitrite) or by the consumption of nitrite.

### Quantification of gaseous nitrogen compounds

For several experiments (mass balance, time course, and one of the SEC runs), ^15^N-labeled sodium nitrite was added to the activity assays instead of non-labeled nitrite, allowing for the detection of all relevant gaseous nitrogen products *via* GC–MS. ^15^N-labeled sodium nitrite was used to differentiate the masses of N_2_O and carbon dioxide (CO_2_) and to distinguish the produced ^30^N_2_ from the residual headspace ^28^N_2_. For GC–MS measurements, 500 μL of the reaction headspace was manually injected using a gas-tight syringe. The GC–MS (GC: Agilent 7890A; MS: Agilent 5975C inert XL MSD with Triple-Axis detector, California, USA) was equipped with a capillary column BXP5 (30 m × 250 μm × 0.25 μm) (SGE Analytical Science, Melbourne, Australia). The GC injector temperature was 250°C, and a split ratio of 50 was applied. Carrier gas was helium, with a column flow rate of 1.63 mL min^−1^. An isothermal program at 40°C (4 min) was used. The *m*/*z* range of 20–100 was scanned. Quantification of N_2_, O_2_, NO, and N_2_O was performed using standard curves generated with the appropriate gas standards. The aqueous concentrations of all gasses in the reaction vials were calculated based on Henry’s law assuming a temperature of 30°C and a pressure of 1 atm. The pressure assumption is sufficiently accurate considering that the vials were brought to atmospheric pressure and that the pressure increase caused by gas production (<5 μL) is negligible. The amounts of gaseous nitrogen compounds in both the gas and liquid phases were quantified, summed, and divided by the liquid phase volume to obtain a nominal liquid concentration, considering the number of nitrogen atoms per molecule (e.g., 2 for N_2_).

### Analytical techniques

Nitrite concentrations were measured either using colorimetric assays with the Griess reagent kit (Molecular Probes, Oregon, USA) following the manufacturer’s instructions (detection limit: 50 μM) or through ion chromatography (detection limit: 5 μM). Ion chromatographic detection of nitrite was done on a Dionex ICS5000 (Thermo Fisher Scientific, Massachusetts, USA) equipped with an IonPac AS18 analytical column (250 × 2 mm) and a UV detector at a wavelength of 210 nm.

Ammonium concentrations were determined using high-performance liquid chromatography–ultraviolet (HPLC–UV) with diethyl ethoxymethylenemalonate (DEEMM) as the derivatizing agent (detection limit: 5 μM). For derivatization, 100 μL of the sample was mixed with 500 μL of reaction buffer containing 7:3 v/v mixture of 50 mM borate buffer (pH 9, adjusted with sodium hydroxide) and methanol and 3 μL of DEEMM, followed by overnight incubation at room temperature in the dark. A 10 μL aliquot of the derivatized sample was injected into an HPLC (UltiMate 3000, Thermo Fisher Scientific, Massachusetts, USA) equipped with a LiChrospher 100 RP-18 (5 μm) LiChroCART 125-4 column using the following gradient program (A: 0.1% v/v formic acid; B: methanol): 20% B from 0 to 1 min, increasing to 100% B from 1 to 5 min, decreasing to 20% B from 5 to 7 min and 20% B from 7 to 9 min. The flow rate was set to 1 mL min^−1^, with the column compartment kept at 35°C. Quantification was conducted by measuring absorbance at 269 nm.

### Shotgun proteomics

Cell extracts and fractionated samples of “*Ca.* Kuenenia stuttgartiensis” strain CSTR1 were analyzed using shotgun proteomics. For fractions after FPLC, between 50 and 100 μL was used as the starting material; for cell extracts and fractionated samples after ultracentrifugation (Supplementary Tables S1, S2), between 10 and 50 μL was used. To achieve accurate label-free quantification and cross-comparison among fractionated samples, glyceraldehyde-3-phosphate dehydrogenase (GAPDH, from *Staphylococcus aureus* MRSA252, NCBI ID: WP_000279414, 336 aa residues) or bovine serum albumin (BSA) were added to each FPLC fraction as internal standards. A working solution of 20 μg ml^−1^ GAPDH or BSA was prepared, of which 5 μL was added to each sample.

Disulfide bonds in protein samples were then reduced with 12-mM DTT (final concentration) for 45 min at 37°C and alkylated with 40-mM iodoacetamide (final concentration) for 45 min at room temperature in the dark, both under gentle shaking. Trypsin digestion was done by adding 5 μL reductively methylated trypsin (0.1 μg μL^−1^, Promega, Wisconsin, USA) to each sample and incubating the samples overnight at 37°C. To stop digestion, formic acid (2% v/v, final concentration) was added to the samples. In the proteomic analyses presented in Supplementary Tables S1, S2, a modified protocol was used where an equal volume of 10% (w/v) sodium deoxycholate was added to each sample before reduction and alkylation, achieving a starting sodium deoxycholate concentration of 5% (w/v). Sodium deoxycholate in samples was brought to 1% (w/v) by adding 100-mM ammonium bicarbonate before trypsin digestion. After trypsin digestion, sodium deoxycholate was precipitated by the added formic acid (2% v/v, final concentration) and was carefully removed by centrifugation (16,000 g for 10 min, twice).

Digested samples were desalted using 100 μL C_18_ Ziptips (Pierce, Thermo Fisher Scientific, Massachusetts, USA), vacuum dried, and reconstituted in 50 μL 0.1% (v/v) formic acid. For measurement, 3–6 μL of each sample was injected into a nano-liquid chromatography with tandem mass spectrometry (LC–MS/MS, Orbitrap) system (Ultimate 3000RSLC, Thermo Fisher Scientific, Massachusetts, USA), following the methods described by Ding and Adrian (2020), except that a 60-min LC gradient instead of the standard 145-min gradient was used for fractionated samples after FPLC. The 60-min gradient was as follows [solution A (0.1% formic acid in water) and solution B (0.08% formic acid in 20% water +80% acetonitrile)]: solution B (%) was 4% for 3 min, increased to 55% from 3 to 43 min, then quickly increased to 90% from 43 to 44 min, held at 90% from 44 to 48 min, decreased to 4% from 48 to 49 min, and held at 4% from 49 to 60 min.

Protein identification was performed using the Proteome Discoverer 2.4 (Thermo Fisher Scientific, Massachusetts, USA) with the SequestHT search engine and the protein database of “*Ca.* Kuenenia stuttgartiensis” strain CSTR1 (National Center for Biotechnology Information [NCBI] GenBank accession number: GCA_011066545.1). As the gene tags from the “*Ca.* Kuenenia” assembly KUST (Strous et al., 2006) have been widely used in the anammox study, we listed gene tags for both our strain CSTR1 and the assembly KUST. In searches of nitrite reductases from potential denitrifiers, two collections of nitrite reductase sequences were used: the first is a sequence collection containing 1,309 protein sequences that were downloaded from UniProtKB/Swiss-Prot using “nitrite reductase” as the search term (accessed November 2024, Supplementary data S1); the second is a compiled collection of NirS/NirK sequences from 4,082 environmental metagenomes (Supplementary data S2) (Pold et al., 2024). These two protein sequence collections were used alongside the protein database of strain CSTR1. False discovery rates at the peptide and protein levels were controlled at 1%. Protein and peptide abundance values were calculated by intensity-based label-free quantification using the Minora node implemented in Proteome Discoverer. For samples fractionated by FPLC, protein quantities were normalized based on the intensity of the internal standards (GAPDH or BSA) in each fraction. The proteomics data were deposited in the ProteomeXchange Consortium *via* the PRIDE partner repository with the dataset identifiers PXD054936, PXD054938, PXD054988, PXD054995, and PXD054997.

## Results

### Optimization of assay conditions for *in vitro* nitrite-reducing activity

Compared to bead beating and freeze/thaw cycles, disruption of cells by French press yielded the highest protein concentration (Supplementary Figure S1); it effectively disrupted the anammoxosome as observed by epifluorescence microscopy. Therefore, cell disruption was subsequently performed using a French press. We then optimized *in vitro* assay conditions for nitrite-reducing activity in “*Ca.* Kuenenia” anammox bacteria based on the assay conditions described by Ferousi et al. (2021). *In vitro* nitrite-reducing activity was highest at pH 6.2 without showing abiotic nitrite reduction (Supplementary Figure S2), which was in agreement with previous studies showing abiotic production of nitric oxide from nitrite with ascorbic acid at low pH (Du et al., 2023). The optimum temperature for activity was 65°C (Supplementary Figure S3). Reactions were nevertheless still incubated at 30°C for practical reasons. Nitrogen gas flushing of headspace (Supplementary Figure S4) or increased headspace-to-liquid volume ratio (Supplementary Figure S5) enhanced nitrite-reducing activity. Both methods reduced the concentration of NO in the assay, thereby alleviating the inhibitory effects of NO on the enzyme, as suggested in a previous report on a different nitrite reductase (Jackson et al., 1991). Subsequent assays were carried out with an ample headspace to liquid volume ratio (0.3-ml reaction volume in 20-ml vials). Ascorbic acid at 1 mM was used as the reductant because abiotic conversion was seen in concentrations higher than 2 mM (Supplementary Figure S6). Finally, the activity was consistently higher in activity tests without shaking than in activity tests that were shaken, so all subsequent activity tests were incubated statically. We have no conclusive explanation for this effect. The optimized recipe for the activity assay was: 200-mM phosphate buffer at pH 6.2 (a mixture of potassium dihydrogen phosphate and dipotassium hydrogen phosphate), 1.0-mM ascorbic acid, 0.2-mM PES, 0.5-mM sodium nitrite, and 1–2% v/v 100× concentrated cells/cell extract (2.5 × 10^10^ cells ml^−1^, protein concentration 2.0 mg ml^−1^) or 16.7% v/v FPLC fractions.

### Mass balance of nitrogen species in the nitrite-reducing activity assay and conversion kinetics

**Figure 1:**
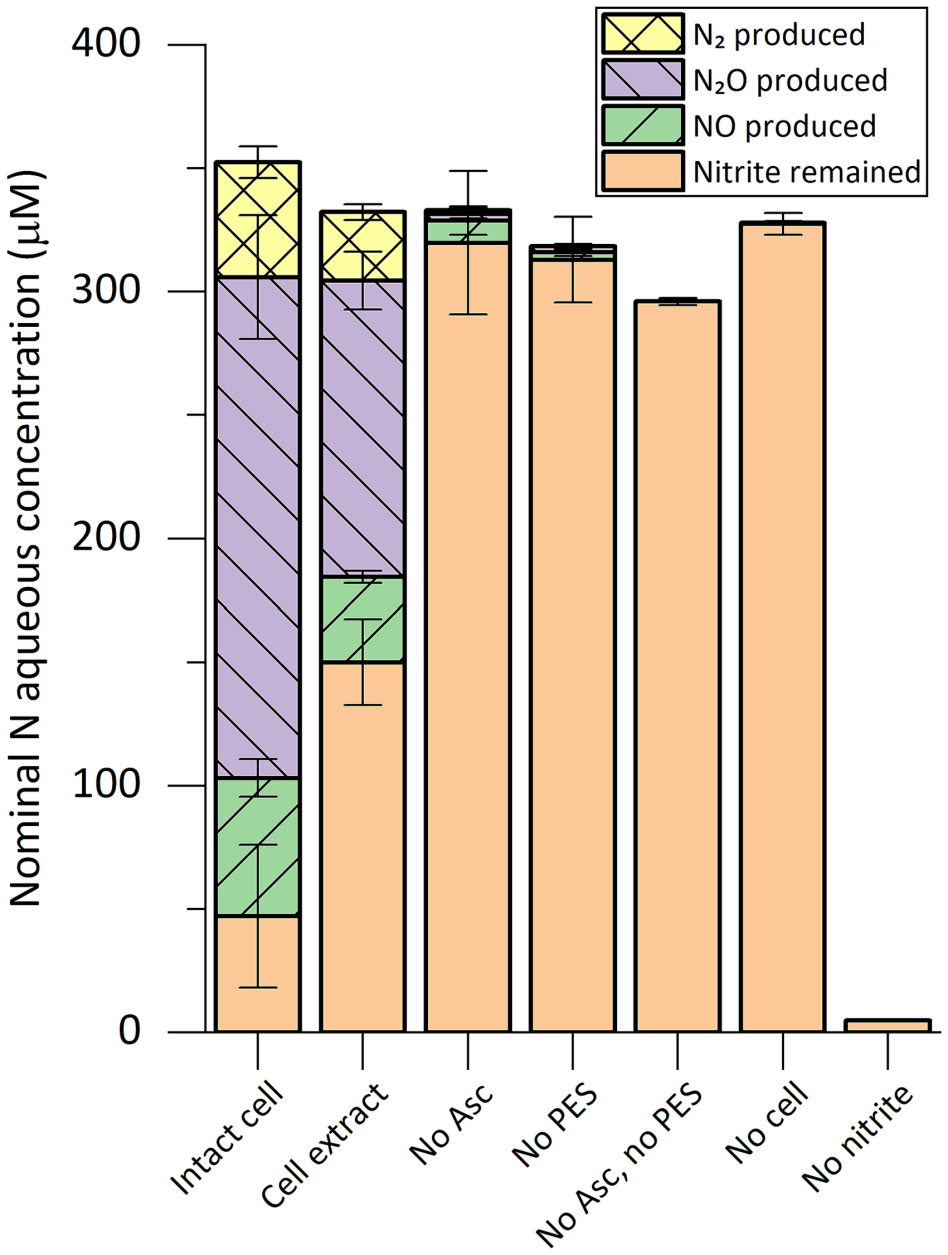
Composition of nitrogen species in *in vitro* nitrite-reducing activity assay vials containing cell extract or intact cells of “*Ca.* Kuenenia stuttgartiensis” strain CSTR1. The nitrogen species are presented as their nominal concentrations in the liquid phase (see “Methods” section). *Intact cell*: reaction containing intact cells of strain CSTR1, buffer, ascorbic acid (Asc), phenazine ethosulfate (PES), and ^15^N-labeled sodium nitrite; *Cell extract*: same as “Intact cell” but using cell extract of strain CSTR1 instead of intact cells; *No Asc*: same as “Cell extract” but containing no ascorbic acid; *No PES*: same as “Cell extract” but containing no PES; *No Asc*, *no PES*: same as “Cell extract” but containing no ascorbic acid or PES; *No cell*: same as “Cell extract” but containing no cell extract; *No nitrite*: same as “Cell extract” but containing no sodium nitrite. The activity assay contained (for condition “Intact cell”/“Cell extract”): 200-mM phosphate buffer (pH 6.2), 1.0-mM ascorbic acid, 0.2-mM PES, 0.33-mM ^15^N-labeled sodium nitrite, 2% v/v 100× concentrated cells/cell extract (2.5 × 10^10^ cells ml^−1^, protein concentration 2.0 mg ml^−1^). Reaction volume: 1 mL. Headspace volume: 9 mL. Incubation time: 2 h. Data shows means of triplicate analysis ± standard deviation.

With the optimized assay conditions, a mass balance analysis of nitrogen species was performed in *in vitro* activity assays where ^15^N-labeled sodium nitrite was added (Figure 1). After 2 h of incubation, NO, N_2_O, and dinitrogen (N_2_) gas were observed in reactions with intact cells or cell extract. The activity required both ascorbic acid as the reductant and PES as the electron mediator, as controls lacking either did not show activity. The traces of activity in the “no ascorbic acid control” might have been supported by residual reducing power in the cell extract. Ammonium concentration was below the detection limit of 5 μM in all reactions. The amount of nitrogen in the remaining nitrite and in the gaseous nitrogen species produced during the assays matched the amount of the initially added nitrite.

**Figure 2:**
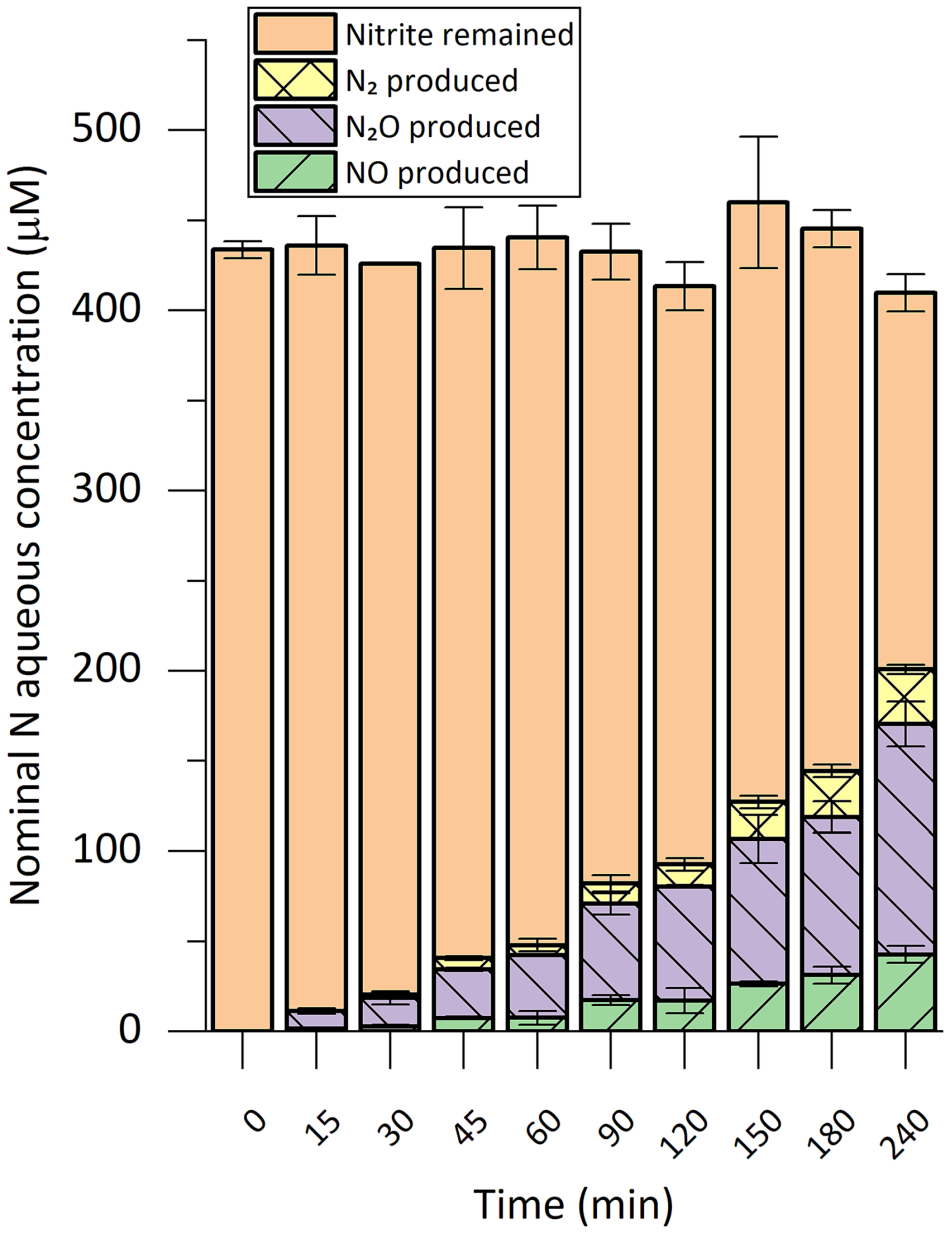
Time course of nitrogen species conversion in *in vitro* nitrite-reducing activity assays with cell extract of “*Ca.* Kuenenia stuttgartiensis” strain CSTR1. The nitrogen species are presented as their nominal concentrations in the liquid phase (see “Methods” section). The activity assay contained 200-mM phosphate buffer (pH 6.2), 1.0-mM ascorbic acid, 0.2-mM phenazine ethosulfate, 0.45-mM ^15^N-labeled sodium nitrite, 1% v/v 100× concentrated cells/cell extract (2.5 × 10^10^ cells ml^−1^, protein concentration 2.0 mg ml^−1^). Reaction volume: 0.3 mL. Headspace volume: 20 mL. Data shows means of triplicate analysis ± standard deviation.

Using the same setup, we performed a time-course analysis of the *in vitro* nitrite-reducing activity in cell extract of strain CSTR1 (Figure 2). Production of gaseous nitrogen compounds was linear in the 4-h incubation time, showing an activity of 0.69 nkat mg^−1^ crude protein.

### Nitrite-reducing activity in fractions separated by size exclusion chromatography

**Figure 3:**
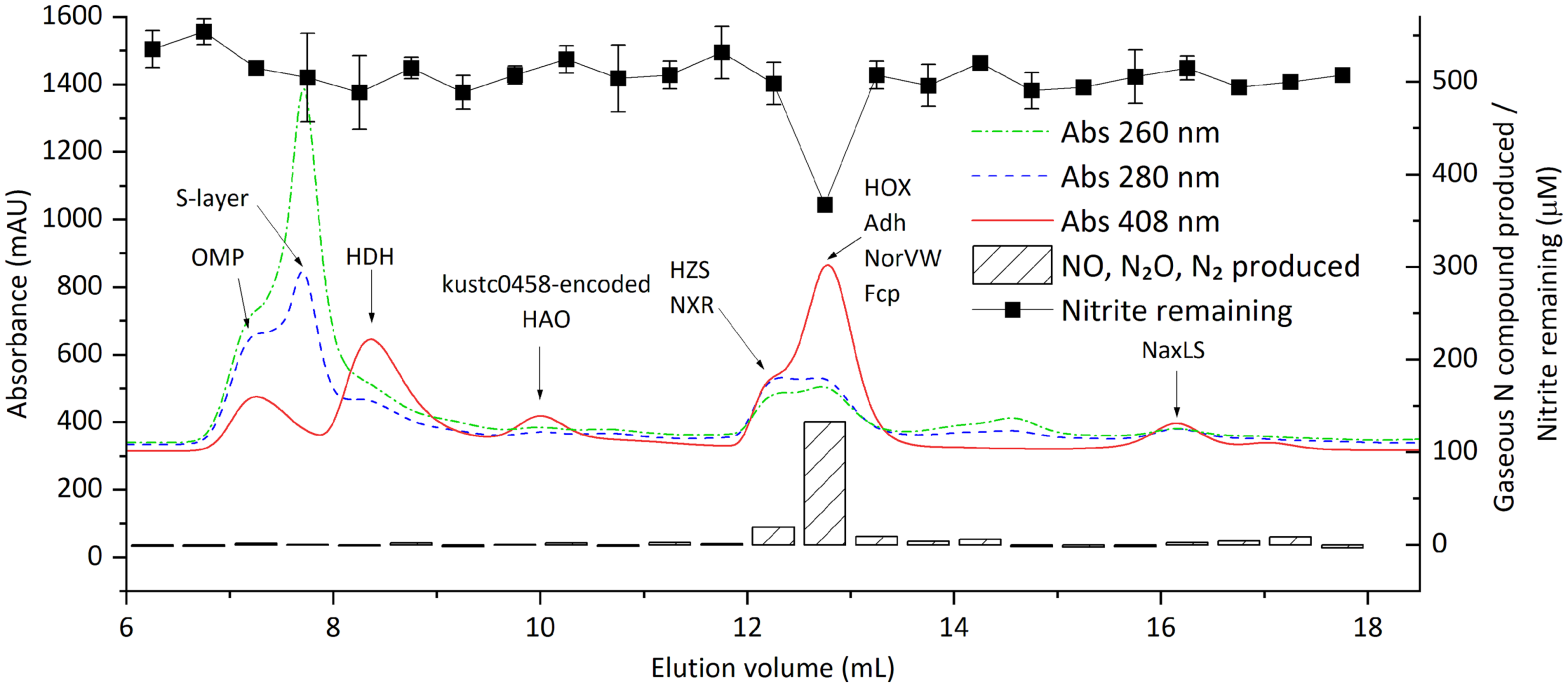
Distribution of proteins and nitrite-reducing activity of “*Ca.* Kuenenia stuttgartiensis” strain CSTR1 after fractionation of crude proteins by size-exclusion chromatography. Shown are the absorptions at 260, 280, and 408 nm (colored lines, left axis) as well as the activity, expressed as the gaseous nitrogen compounds produced after overnight incubation (striped bars, right axis) and the remaining nitrite (filled squares, right axis) in 0.5-ml fractions. Gaseous nitrogen compounds produced are presented as their total nominal concentrations in the liquid phase (see “Methods” section). Proteins of interest, detected by mass spectrometry in the fractions, are labeled on the fraction in which they were detected. The biological material for this chromatography was 100 μL cell extracts from 100× concentrated cells (2.5 × 10^10^ cells ml^−1^, protein concentration 2.0 mg ml^−1^). The activity assay contained 20-mM MOPS buffer at pH 7.2, 0.5-mM ascorbic acid, 1.0-mM phenazine methosulfate, 0.5-mM sodium nitrite, and 16.7% v/v eluted fraction. This experiment (activity assays and proteomics of the fractions) was done in an early stage when the activity assay conditions were not yet optimized, and therefore required an overnight incubation to observe adequate signals. Later experiments with optimized assay conditions saw a similar distribution of nitrite-reducing activity around an elution volume of 13 mL (Supplementary Figure S7). Detailed protein mass spectrometric results are listed in Supplementary Table S3.

To associate protein identities with nitrite-reducing activity, we natively separated the proteome of strain CSTR1 using size exclusion chromatography (SEC). Fractions of 0.5 mL between elution volumes of 6 and 18 mL (corresponding to protein sizes larger than ~10 kDa) were collected. The collected fractions were then subjected to both shotgun proteomics and nitrite-reducing activity assays (Figure 3; Supplementary Figure S7; Supplementary Table S3). In total, 1,661 proteins were identified and quantified. Proteins known to form complexes were found to coelute, including three subunits of hydrazine synthase (HZS) (peak fraction F13 at 12.0–12.5 mL), three subunits of nitrite:nitrate oxidoreductase (NXR) (peak fraction F13 at 12.0–12.5 mL), the HAO encoded by KsCSTR_49490 (kustc0458) and its redox partner protein encoded by KsCSTR_49500 (corresponding to kustc0457) (peak fraction F8 at 9.5–10.0 mL), the nitric oxide-binding heterodimeric cytochrome *c* complex [NaxL, encoded by KsCSTR_40390 (corresponding to kusta0087), and NaxS, encoded by KsCSTR_40380 (corresponding to kusta0088)] (peak fraction F21 at 16.0–16.5 mL).

The nitrite-reducing activity was consistently seen in the cytochrome-rich peak (indicated by absorbance at 408 nm) at around 12.8 mL (fraction F14, 12.5–13.0 mL), corresponding to a molecular weight of 150–200 kDa. GC–MS analysis confirmed stoichiometric production of NO, N_2_O, and N_2_ in the active fractions (Figure 3). Recovery of activity in all eluted fractions reached up to 70% of that in the injected cell extract, indicating that the majority of the activity was retained during SEC.

Among a total of 102 proteins that showed their highest amount in the fraction F14, the annotated hydroxylamine dehydrogenase [HOX, encoded by KsCSTR_43280 (kustc1061)] had the highest abundance, with an intensity of 1.6 × 10^10^ as quantified from precursor mass peaks. Other proteins that were of potential interest based on their annotation included: (1) NADP-dependent isopropanol dehydrogenase (Adh, encoded by KsCSTR_21150 (corresponding to kuste3626), the precursor peak intensity of 1.6 × 10^9^); (2) a protein annotated as nitrite reductase electron transfer 4Fe-4S subunit (Fcp, encoded by KsCSTR_46210 (corresponding to kustc0775), the precursor peak intensity of 4.2 × 10^8^), and (3) nitric oxide detoxifying flavorubredoxin (NorVW, encoded by KsCSTR_15610 (corresponding to kuste3160), precursor mass intensity of 2.1 × 10^7^). Notably, the fractions F8 and F9 between 9.5–10.5 mL containing the HAO encoded by KsCSTR_49490 (kustc0458) did not show discernible nitrite-reducing activity, in contrast to what has been reported previously (Ferousi et al., 2021). Moreover, the canonical nitrite reductase NirS in the genome of CSTR1 did express, albeit with low abundances, with fraction F13 (12.0–12.5 mL) containing the highest abundance (1.1 × 10^7^), followed by fraction F4 (7.5–8.0 mL) (8.9 × 10^6^).

**Figure 4:**
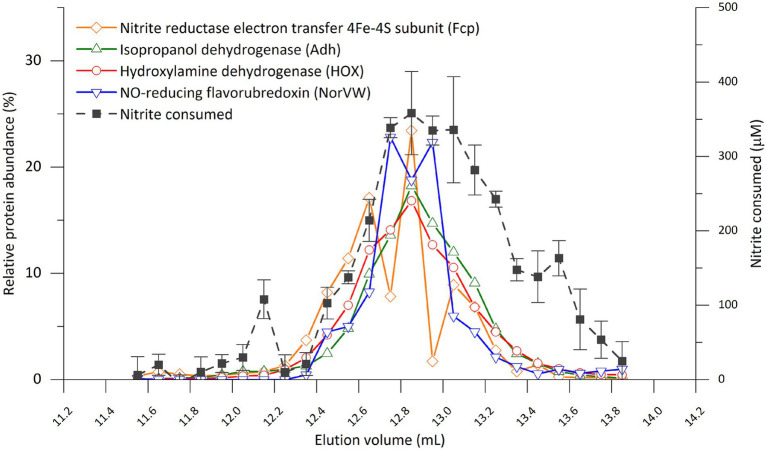
Distribution of nitrite-reducing activity and selected proteins from of “*Ca.* Kuenenia stuttgartiensis” strain CSTR1 in fractions separated by size-exclusion chromatography (0.1 mL per fraction between 11.5 and 13.9 mL elution volume). The four proteins were selected based on their abundance or annotation as stated in the “Results” section. The full list of proteins and their distributions can be found in Supplementary Table S4. Abnormal abundances for Fcp in fractions 12.7–12.8 and 12.9–13.0 mL were due to ionization problems in the mass spectrometer that affected an abundantly detected peptide for this protein, while a repetition of the experiment showed a smoother trend (Supplementary Figure S8). The activity assay contained 200-mM phosphate buffer (pH 6.2), 0.5-mM ascorbic acid, 0.2-mM phenazine ethosulfate, 0.5-mM sodium nitrite, and 16.7% v/v eluted fraction. Incubation time: 4 h. Activity data shows means of triplicate analysis ± standard deviation.

To reveal the correlation between activity and protein distribution at finer resolution, we performed SEC separation of the cell extract again and divided the peak showing activity into smaller fractions (0.1 mL) (Figure 4; Supplementary Figure S8; Supplementary Table S4). All four proteins mentioned above still coeluted with the nitrite-reducing activity except that the peak of NorVW eluted slightly (0.2 mL) earlier than that of the activity in one of the runs (Supplementary Figure S8). There were discrepancies between the nitrite-reducing activity and protein abundance in Figure 4 from 13 mL onward, while another run, as presented in Supplementary Figure S8, showed a more consistent trend.

Based on the above results, we concluded that the nitrite-reducing activity under *in vitro* conditions was catalyzed by one or several proteins in the fraction F14 (elution volume 12.5–13.0 mL), most likely the HOX, Adh, Fcp, or NorVW. The HAO encoded by KsCSTR_49490 (kustc0458) which was previously suspected as the nitrite reductase under physiological conditions (Ferousi et al., 2021) did not contribute significantly to the nitrite-reducing activity under *in vitro* conditions.

### Nitrite-reducing activity in fractions separated by anion exchange chromatography

**Figure 5:**
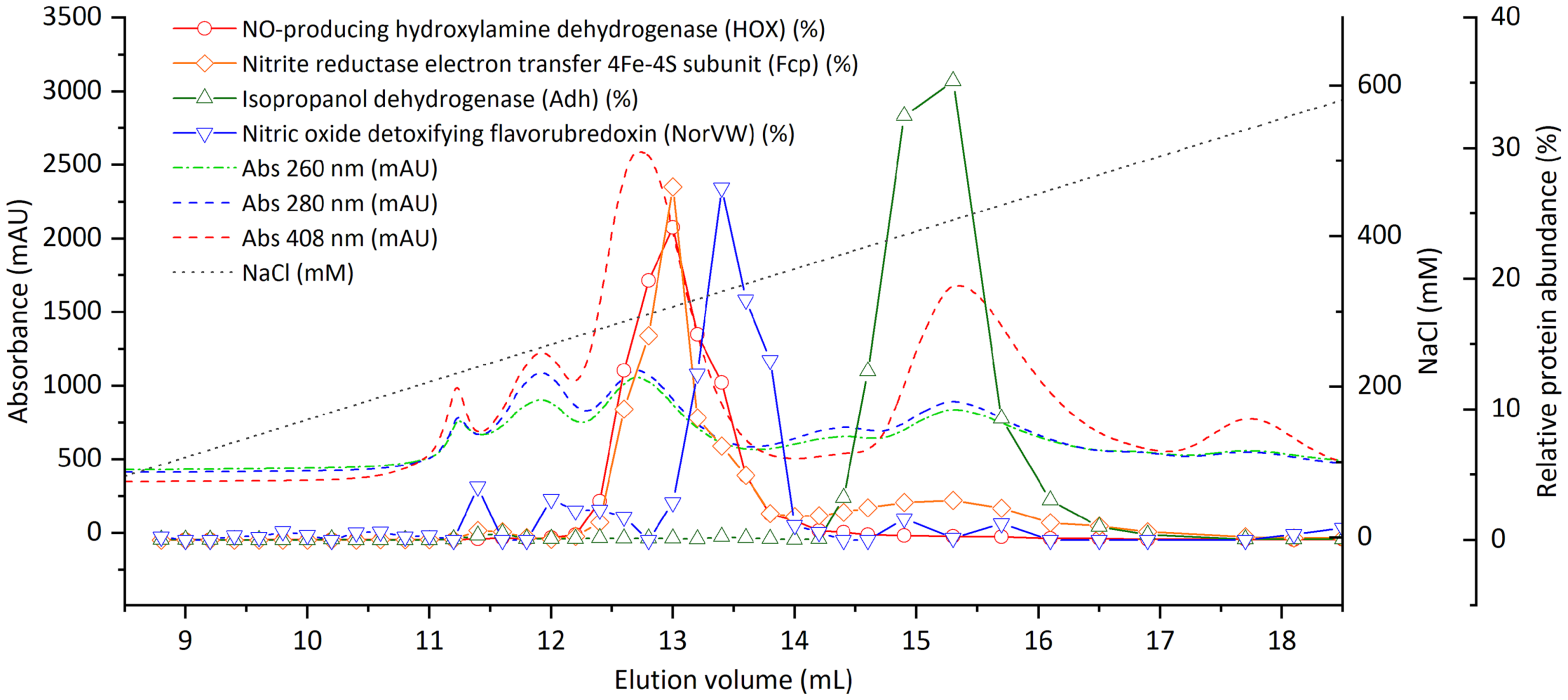
Distribution of selected proteins of “*Ca.* Kuenenia stuttgartiensis” strain CSTR1 after fractionation by anion exchange chromatography. The four proteins were selected based on their abundance or annotation, as stated in the “Results” section. The complete list of proteins and their distributions can be found in Supplementary Table S5. Shown are the absorptions at 260, 280, and 408 nm (colored dashed lines, left axis) and the relative abundance of proteins of interest (colored solid lines, right axis) in 0.2–0.4 mL fractions. The biological material for this chromatography was 500 μL cell extracts from 100× concentrated cells (2.5 × 10^10^ cells ml^−1^, protein concentration 2.0 mg ml^−1^).

We then separated the proteome of strain CSTR1 by anion exchange chromatography (AEC), to see if the suspected proteins still co-elute with the nitrite-reducing activity (Figure 5; Supplementary Table S5; Supplementary Figure S9).

On AEC, Fcp still coeluted with HOX at a NaCl concentration of about 300 mM (Figure 5). NorVW eluted at a slightly higher NaCl concentration of 330 mM, and Adh eluted at 420-mM NaCl.

In contrast to SEC where nitrite-reducing activity eluted as a single peak, a low recovery of nitrite-reducing activity (up to 30% for all fractions combined) was constantly observed on AEC (Supplementary Figure S9). The fractions containing HOX (at 300-mM NaCl) still showed moderate nitrite-reducing activity, but fractions at higher NaCl concentrations also showed nitrite-reducing activity (Supplementary Figure S9). To ensure that the salt in the fractions did not interfere with nitrite-reducing activity, we collected the AEC fraction containing HOX, and separated it using SEC. However, no nitrite-reducing activity was observed, even though the cytochrome-rich peak was clearly visible at the expected position in the SEC for HOX (data not shown).

### Fractionation of nitrite-reducing activity using ultracentrifugation and ultrafiltration

Since fractionation by SEC and AEC did not show a clear protein candidate responsible for nitrite reduction, we performed fractionation of the proteome of strain CSTR1 using ultracentrifugation and ultrafiltration. The hypothesis was that we could understand the cellular localization and molecular size ranges of the nitrite-reducing activity through such fractionation and could simplify the proteome composition for subsequent FPLC separation. Cell extract of strain CSTR1 was fractionated by ultracentrifugation for 3 h at 130,000 g. Apart from the yellowish supernatant (cytosolic fraction) and the tight dark reddish pellet (membrane fraction), we observed an additional layer right above the membrane pellet that appeared as dark red liquid (Supplementary Figure S10). Nitrite-reducing activity was found to be predominantly (60–85%) located in this intermediate fraction (Supplementary Figure S11).

Proteomics analysis of the three fractions after ultracentrifugation (Supplementary Table S1) yielded 1,698 proteins. Of them, 877 had their highest abundance in the membrane fraction, including the outer membrane protein (encoded by KsCSTR_06200 (corresponding to kustd1878), 99.6% in membrane fraction), the most abundant nitrite transporter (encoded by KsCSTR_14610 (corresponding to kuste3055), 99.7%), hydrazine dehydrogenase (99.6%), three subunits of HZS (93.7–96.5%), three subunits of NXR (86.9–94.9%) as well as proteins encoded by genes in the same cluster KsCSTR_07880–KsCSTR_07950 (corresponding to kustd1705–kustd1712, 84.0–99.5%). A total of 589 proteins had their highest abundance in the cytosolic fraction, including NaxLS (encoded by KsCSTR_40380 (corresponding to kusta0088), KsCSTR_40390 (corresponding to kusta0087), 55.3–62.9% in the cytosolic fraction), two blue copper proteins (encoded by KsCSTR_08010 (corresponding to kustd1699), and KsCSTR_07870 (corresponding to kustd1713), 65.4–69.4%). The remaining 232 proteins had their highest abundance in the intermediate fraction, including Adh (42.9% in the intermediate fraction, 40.3% in the cytosolic fraction). Although HOX, NorVW, and Fcp were abundant in the membrane fraction (57.2, 60.4, and 54.3%, respectively), they were also detected in a significant share in the intermediate fraction (42.0, 35.3, and 33.0%, respectively). Besides, a clear correlation between distribution after ultracentrifugation and distribution in SEC fractions was observed (Supplementary Figure S12), indicating that the separation between the membrane fraction and the other two fractions was protein-size dependent, that is, larger proteins ended up more likely in the membrane fraction.

**Figure 6:**
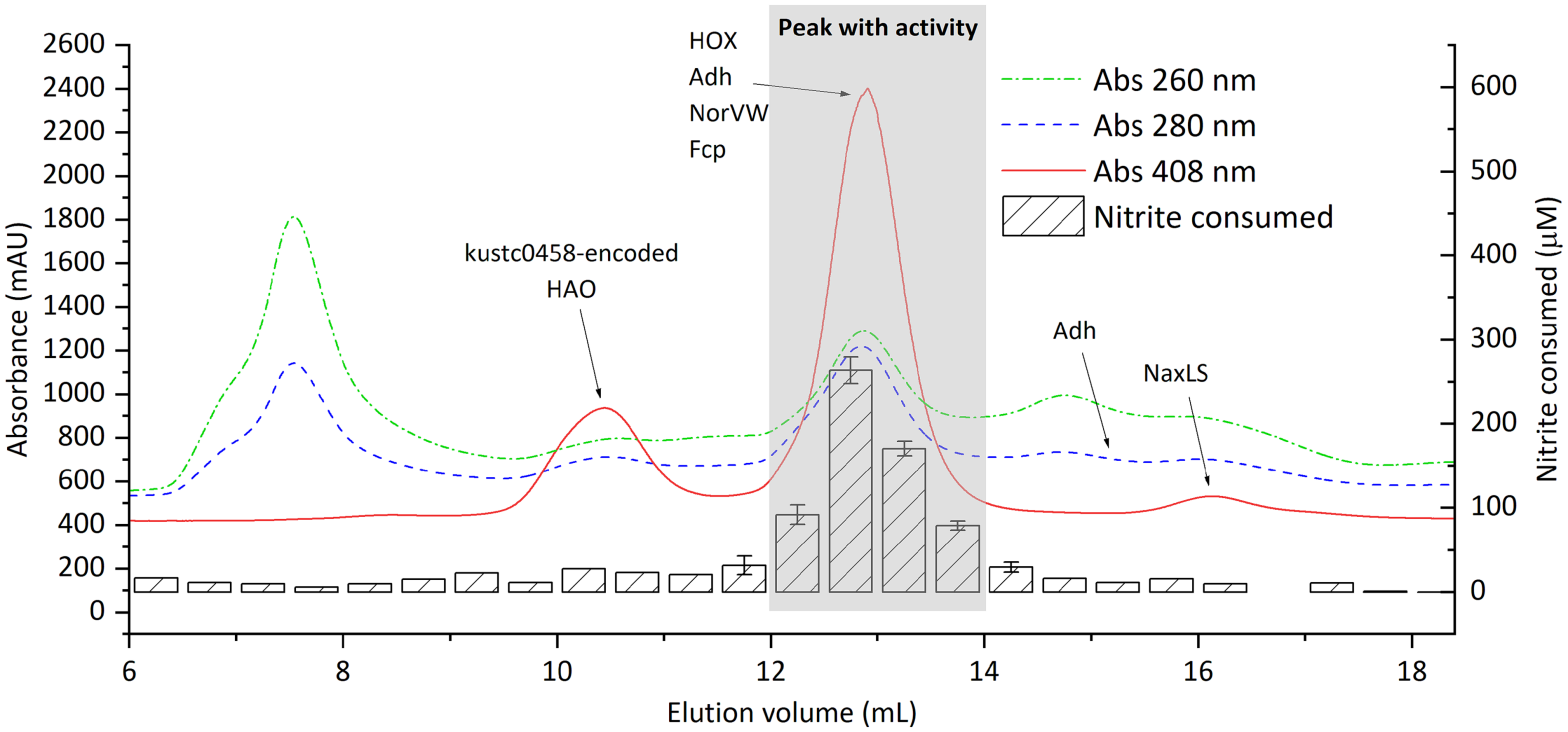
Distribution of proteins and nitrite-reducing activity of “*Ca.* Kuenenia stuttgartiensis” strain CSTR1 after fractionation by size-exclusion chromatography. Shown are the absorptions at 260, 280, and 408 nm (colored lines, left axis) and the activity, expressed as the concentration of nitrite consumed after 3 h of incubation (striped bars, right axis) in 0.5 mL fractions. Proteins of interest, detected by mass spectrometry in the fractions, are labeled on the fraction in which they were detected. Adh was separated into two peaks, whose positions on the elution curve are labeled (at 12–14 and 15–15.5 mL elution volume). The biological material for this chromatography was obtained from the >100 kDa fraction after ultracentrifugation (taking the intermediate fraction) and ultrafiltration (Supplementary Figure S13). The activity assay contained 200-mM phosphate buffer (pH 6.2), 1.0-mM ascorbic acid, 0.2-mM phenazine ethosulfate, 0.5-mM sodium nitrite, and 16.7% v/v eluted fraction. Detailed protein mass spectrometric results are listed in Supplementary Table S2.

The activity-containing intermediate fraction after ultracentrifugation was then subjected to ultrafiltration with molecular filters (cutoff size 100 and 3 kDa) (Supplementary Figure S13). The activity was repeatedly found in the retained fraction in the 100 kDa filter, that is, in fractions with size above 100 kDa. This retained fraction was then separated using SEC, after which activity assays and proteomics were performed (Figure 6). Again, activity was found in fractions eluting between 12 and 14 mL, as had been observed when the crude extract was directly separated by SEC (Figure 3). A total of 29 proteins exhibited a distribution that matched the distribution of nitrite-reducing activity distribution. Among these, all the four proteins of interest discussed in the section “nitrite-reducing activity in fractions separated by size exclusion chromatography” were included (HOX, Fcp, Adh, and NorVW), with HOX being the most abundant protein by far (Supplementary Table S6).

## Discussion

The presence of nitric oxide as a key intermediate in “*Ca.* Kuenenia” anammox has been experimentally confirmed (Kartal et al., 2011). However, the identity of the enzyme that converts nitrite to nitric oxide remains unclear. In this study, we established and optimized a highly sensitive *in vitro* assay for the detection of nitrite reductase from protein preparations of “*Ca.* Kuenenia stuttgartiensis” and used it to detect nitrite-reducing activity in fractions obtained from several fractionation methods, including size exclusion chromatography, anion exchange chromatography, ultracentrifugation, and ultrafiltration. The results indicated that several proteins that were not previously associated with nitrite reduction may be involved in the reduction of nitrite to NO.

### Nitrite-reducing activity was observed *in vitro* with nitric oxide as the main product

As a prerequisite for the detailed analysis of nitrite reduction, we optimized the *in vitro* activity assay for highly sensitive detection and quantification of nitrite reduction. The requirement of an acidic pH is consistent with previous findings that suggested a pH of 6.3 inside the anammoxosome where nitrite reduction occurs (van der Star et al., 2010). With the established activity assay, we sensitively and reproducibly detected and quantified nitrite-reducing activity in cell extract and fractions of “*Ca.* Kuenenia stuttgartiensis” strain CSTR1 [except for AEC fractions where a low recovery of activity was observed (Supplementary Figure S9)]. The nitrite-reducing process was nitrogen balanced (Figures 1, 2), with no ammonium generated, indicating that no significant activity of dissimilatory nitrite reduction to ammonium (DNRA) was present (Kroneck, 2022). The products of the nitrite-reducing activity were confirmed to be NO, along with N_2_O and N_2_ gas. N_2_O and N_2_ were likely formed by further reduction from NO, either enzymatically or abiotically. Two processes may be responsible for the further conversion of NO: on the one hand, the excess amount of ascorbate in the reaction might further reduce NO to N_2_O and N_2_ gas; on the other hand, nitric oxide reductases [e.g., NorVW and the protein encoded by KsCSTR_15610 (kuste3160)] might also participate in such conversion. In addition to NorVW, several HAOs (non-anammox origin) have been reported to reduce NO to N_2_O (Kostera et al., 2008). Finally, enzymes from denitrifiers that probably existed in the microbial community may also be involved in the conversion of NO. Regardless of how the conversion of NO took place, the concentration of NO was effectively reduced, with only 0.22 μM of NO remaining in the aqueous phase after 4 h of incubation (calculated based on Henry’s law). The relatively low NO concentration may have helped in sustaining nitrite-reducing activity *in vitro*, considering NO is inhibitory to many proteins through nitrosylation or oxidizing protein thiols (Brown, 1999; Ceccaldi et al., 2016).

### Our experimental evidence suggests that the HAO encoded by KsCSTR_49490 (kustc0458) is not the key enzyme responsible for nitrite reduction under physiological conditions, nor are the canonical nitrite reductases

From our experiments, we obtained clear evidence that the HAO encoded by KsCSTR_49490 (kustc0458) is not strongly contributing to the nitrite reduction step in the anammox metabolism in “*Ca.* Kuenenia stuttgartiensis” at least under *in vitro* conditions, which is in contrast to what was proposed earlier (Ferousi et al., 2021). This conclusion is based on several results: first, when using reduced PES as the electron donor, the measured nitrite-reducing activity (0.69 nkat mg^−1^ crude protein) in this study was two orders of magnitude higher than that of the purified protein encoded by kustc0458 (KsCSTR_49490) (0.52 mU mg^−1^ protein = 8.7 pkat mg^−1^ protein). Therefore, the activity measured with the protein encoded by kustc0458 (KsCSTR_49490) might be only a side activity. Second, in SEC separation of the proteome of strain CSTR1, we reproducibly found no noticeable activity in the fractions in which the protein encoded by KsCSTR_49490 (kustc0458) was detected (at about 10 mL elution volume). In comparison, activity was detected only at around 12.8 mL elution volume. This indicates that the enzyme(s) that catalyze nitrite reduction are among the proteins eluting at 12.8 mL, but not the protein encoded by KsCSTR_49490 (kustc0458). Hence, the physiological roles of the proteins encoded by KsCSTR_49490 (kustc0458) and several other HAO genes, remain unclear. The canonical nitrite reductase NirS probably also did not play a significant role in nitrite reduction in strain CSTR1, as its distribution after SEC separation did not agree with that of the nitrite-reducing activity (Supplementary Table S3). We also examined the presence of nitrite reductases listed in the public database in our proteomics datasets, including 1,309 nitrite reductases listed in UniProt/Swiss-Prot and a collection of NirS/NirK sequences compiled by Pold et al. (2024). Several NirS and NirK proteins were identified at low abundances (Supplementary Table S7), but none of their abundance distributions aligned with that of the nitrite-reducing activity (data not shown).

### The role of hydroxylamine dehydrogenase HOX (encoded by KsCSTR_43280/kustc1061) in nitrite reduction

Several proteins reproducibly eluted at around 12.8 mL in SEC, including the NO-producing hydroxylamine dehydrogenase HOX [encoded by KsCSTR_43280 (kustc1061)] which was one of the most abundant proteins at this elution position. HOX (60.2 kDa) forms a trimer (Maalcke et al., 2014), which agrees with the elution position (even after considering complexation with the 15.8 kDa Fcp as discussed below). HOX was reported to mainly catalyze oxidative reactions, for example, the conversion of hydroxylamine to NO, releasing three electrons (Maalcke et al., 2014). This reaction can be physiologically important, considering that hydroxylamine may leak during hydrazine synthesis by HZS in “*Ca.* Kuenenia.” However, since HOX is the third most abundant protein (after HZS subunits and HDH) in the proteome of “*Ca.* Kuenenia” (Supplementary Table S1), it is a matter of debate, whether hydroxylamine detoxification alone would explain its high expression level. Notably, HOX was also reported to have nitrite-reducing activity (0.18 U mg^−1^ protein = 3.0 nkat mg^−1^ protein) with reduced methyl viologen as the electron donor (Maalcke et al., 2014). Such activity is not negligible, especially when multiplied by the high abundance of the protein in the proteome. Interestingly, a copper-containing dissimilatory nitrite reductase (NirK) in an ammonia-oxidizing archaeon was also found to have hydroxylamine to NO activity (Kobayashi et al., 2018), indicating that it might be a common feature for a group of enzymes to possess both reductive and oxidative NO-producing activities (i.e., nitrite reduction and hydroxylamine oxidation).

A series of additional pieces of evidence is in agreement with the hypothesis that HOX is a nitrite reductase candidate. First, the expression of HOX was significantly downregulated as seen in its transcripts and protein amount in a NO-fed anammox reactor, compared to the nitrite-fed anammox reactor as a control (Hu et al., 2019). Such downregulation even speaks against the significant role of HOX as hydroxylamine dehydrogenase since the leakage of hydroxylamine should remain at a similar level regardless of whether NO or nitrite is supplied to the anammox bacteria. Second, as with other key enzymes in the anammox metabolism, HOX was found to be located inside the anammoxosome, where the anammox reactions, including nitrite reduction, take place (Kartal et al., 2013; Kartal et al., 2012). Third, the HOX-encoding gene was found to be moderately conserved sharing >93% translated amino acid identity among “*Ca.* Kuenenia” genomes including the recently discovered “*Ca.* Kuenenia hertensis” (Yang et al., 2021) and at least 55% identity with “*Ca.* Brocadia,” “*Ca.* Jettenia,” and “*Ca.* Scalindua” (Okubo et al., 2021). Since all anammox bacteria are known to possess nitrite-reducing activity, they must encode a nitrite reductase. The conservation of the HOX-encoding gene across all anammox genomes supports the hypothesis that it may serve as a common nitrite reductase gene.

### Multiple proteins may be involved in nitrite reduction

Although the results from SEC hint at a role of HOX as the nitrite reductase in “*Ca.* Kuenenia,” AEC runs were inconclusive: activities of AEC fractions were all much lower than those of SEC fractions, and the already lower activities were distributed into several peaks after AEC (Figure 5; Supplementary Table S5; Supplementary Figure S9). It is possible that the enzyme catalyzing nitrite-reducing activity lost much of its activity during AEC separation. Another possibility is that multiple proteins were involved in nitrite-reducing activity, and these proteins co-eluted or formed a complex in SEC but were separated in AEC. It is, therefore, important to interpret the results from AEC with care.

Based on their distribution in SEC and annotations, three proteins, in addition to HOX, were identified as interesting candidates: Fcp [encoded by KsCSTR_46210 (kustc0775)], NorVW [encoded by KsCSTR_15610 (kuste3160)], and Adh [encoded by KsCSTR_21150 (kuste3626)]. These four proteins eluted precisely at the elution position of the activity, in the 0.5-ml and even 0.1-ml resolution SEC fractionation with crude cell extracts (Figure 4; Supplementary Figure S8). The four proteins also coeluted with the activity in the SEC run with pre-fractionated cell extracts (ultracentrifugation and ultrafiltration) (Supplementary Table S6). However, we emphasize that HOX, Fcp, NorVW, and Adh are not an exhaustive list of candidate proteins potentially involved in nitrite reduction, and additional yet unidentified proteins may also play a role.

Regarding the identified proteins, Fcp, in particular, co-eluted with HOX in both SEC and AEC runs (Figure 5), and might form a complex with HOX. Fcp has a predicted size of 15.8 kDa, and is expected to contain an [FeS]-cluster-containing hydrogenase component 2 motif (amino acid position 9–141, E-value: 1.82 × 10^−22^) when searched against the NCBI Conserved Domain Database (Marchler-Bauer et al., 2013). If Fcp indeed forms a complex with HOX, it could be involved in electron transfer to HOX for nitrite reduction. Notably, Fcp is encoded by one of the most conserved genes among anammox genomes (Speth, 2015) and shares >94% amino acid sequence identity among “*Ca.* Kuenenia” genomes (including “*Ca.* Kuenenia hertensis”), 80–90% sequence identity with the homologs in “*Ca.* Brocadia” genomes, and 60–70% sequence identity with the homologs in “*Ca.* Scalindua” genomes.

NorVW (predicted size of 44.5 kDa) coeluted with HOX in SEC but was partially separated from HOX in AEC. It may form a trimer or tetramer if not being in complex with other proteins. NorVW catalyzes the reduction of NO to N_2_O in *Escherichia coli* (Gardner et al., 2002). Therefore, it is tempting to speculate that the presence of nitric oxide-reducing activity by NorVW alleviated inhibition by nitric oxide, thereby sustaining nitrite-reducing activity. However, we are reluctant to draw such a conclusion as (1) NorVW and the highly suspected candidate HOX still partially overlapped in their elution profiles in AEC (Figure 5) but the majority of activity was still lost, and (2) it would be wasteful to divert NO, the valuable intermediate inside anammox cells, from the key anammox reaction (condensation with ammonium to form hydrazine). Since NorVW has no export signature as predicted from SignalP (version 6.0) (Teufel et al., 2022), its physiological role is probably detoxification of NO which diffuses through the anammoxosome membrane into the cytoplasm. The fact that NorVW was constantly expressed under normal and inhibiting conditions supports such a role (Yan et al., 2020). Another possibility is that NorVW is transported as a complex together with another protein into the anammoxosome. NorVW shares >85% amino acid sequence identity among “*Ca.* Kuenenia” genomes (including “*Ca.* Kuenenia hertensis”), 70–80% sequence identity with homologs in “*Ca.* Brocadia” and “*Ca.* Jettenia” genomes, but is not encoded in the genomes of “*Ca.* Scalindua.”

Adh (predicted size of 37.5 kDa) forms tetramers (150 kDa) according to the literature (Karlsson et al., 2003), which matches its elution position at 12.5–13.0 mL in SEC. The Adh tetramer appeared to partially dissociate during SEC after fractionation by ultracentrifugation or ultrafiltration (Supplementary Table S2; Figure 6), as a second peak appeared at 15.0–15.5 mL where no nitrite-reducing activity was detected. The Adh-encoding gene appears to be only conserved within the “*Ca.* Kuenenia stuttgartiensis” species (100%) while it is not encoded in the draft genome of “*Ca.* Kuenenia hertensis.”

### Protein localization and complex formation as revealed by ultracentrifugation and SEC

In this study, we fractionated and analyzed proteins in cell extracts of “*Ca.* Kuenenia” using ultracentrifugation and SEC. The majority of proteins that were reported to coelute in the complexome analysis with membrane proteins of “*Ca.* Kuenenia” (de Almeida et al., 2016) also coeluted on SEC in this study (Supplementary Table S3). Expression levels of the most abundant proteins were also consistent with those reported by de Almeida et al. (2016) (Supplementary Table S1). Since our SEC data were obtained from whole cell extracts instead of membrane fractions, many additional co-elution patterns of cytosolic proteins were found, including NaxL [encoded by KsCSTR_40390 (kusta0087)] with NaxS [encoded by KsCSTR_40380 (kusta0088)], the aforementioned HAO [encoded by KsCSTR_49490 (kustc0458)] with its redox partner [encoded by KsCSTR_49500 (kustc0457)], heme biosynthesis proteins [encoded by KsCSTR_41990 (corresponding to kustc1192) with the protein encoded by KsCSTR_41980 (corresponding to kustc1193)], as well as the aforementioned HOX with Fcp.

Although proteins in early-eluting SEC fractions (having larger sizes or forming complexes) tended to be associated with the membrane fraction after ultracentrifugation (Supplementary Figure S12), there were, nevertheless, proteins that were primarily found in the membrane fraction but eluted relatively late on SEC. Examples include the ammonium transporter [encoded by KsCSTR_00670 (corresponding to kustc0381)] (94% in the membrane fraction, eluted at 12.0–12.5 mL on SEC), hydrazine synthase subunits HZS (average 95% in the membrane fraction, eluted at 12.0–12.5 mL on SEC), and nitrite:nitrate oxidoreductase subunits NXR (average 92% in the membrane fraction, eluted at 12.0–12.5 mL on SEC). Some of these proteins, for example, the ammonium transporter, were probably buried in the cell membrane and got only detached during SEC runs. Some other proteins, for example, HZS and NXR, were reported as non-membrane-bound proteins in the anammoxosome. NXR forms a tubular structure of several hundred nanometers long inside the anammoxosome (Chicano et al., 2021), the assembly of which was assisted by NXR-T (84% in the membrane fraction). Such tubular architecture probably led to its precipitation in the membrane fraction but subsequently disintegrated during the SEC run. The exclusive distribution of HZS in the membrane fraction was unexpected, considering its heterotrimer size of 170 kDa (or 197.3 kDa when forming a complex with NaxLS) (Dietl et al., 2015). It is possible that HZS had weak interactions with the membrane or a large cellular structure and co-precipitated during ultracentrifugation. Finding the exact cause may help elucidate the electron transfer process in the anammox metabolism.

## Conclusion

This study advances our understanding of the enzymatic processes involved in nitrite reduction in “*Ca.* Kuenenia stuttgartiensis.” By establishing a sensitive *in vitro* assay, we detected nitrite-reducing activity in “*Ca.* Kuenenia” cell extracts, as well as fractionated samples, and confirmed nitric oxide as the main product. Our findings contrast with earlier attributions of nitrite-reducing roles to the HAO encoded by KsCSTR_49490/kustc0458, highlighting the need to reassess its physiological role. Instead, our results suggest a pivotal role for hydroxylamine dehydrogenase HOX (encoded by KsCSTR_43280/kustc1061) in nitrite reduction, supported by its distribution in SEC and ultracentrifugation/ultrafiltration fractions containing nitrite-reducing activity as well as its conservation across anammox genomes. Furthermore, we suggest a potential involvement of other proteins, namely Fcp, NorVW, and Adh, in nitrite reduction in “*Ca.* Kuenenia.” This work provides a foundation for future research on the molecular mechanisms involved in nitrite reduction in anammox metabolism.

## Data Availability

The datasets presented in this study can be found in online repositories. The names of the repository/repositories and accession number(s) can be found in the article/Supplementary material.
