# Supplementary material for: Fractionating proteins with nitrite-reducing activity in “Candidatus Kuenenia stuttgartiensis” strain CSTR1
Source: Front Microbiol. 2025 Feb 26;16:1483703. doi: 10.3389/fmicb.2025.1483703 (PMC11897245; doi:10.3389/fmicb.2025.1483703)
Supplement: Supplementary file 1 [file Supplementary_file_1.zip › Supplementary material.pdf]

Supplementary Materials for "Fractionating proteins with nitrite-reducing activity in "*Candidatus* Kuenenia stuttgartiensis" strain CSTR1"

Emea Okorafor Ude[1], Pranathi Sure[1], Rimjhim Rimjhim[1], Lorenz Adrian[1,2], Chang Ding[1,*]

[1]Helmholtz Centre for Environmental Research – UFZ, Department of Molecular Environmental Biotechnology, Leipzig, Germany

[2]Chair of Geobiotechnology, Technische Universität Berlin, Berlin, Germany

Running title: Nitrite-reducing activity in anammox bacteria

*To whom correspondence should be addressed: Chang Ding, Helmholtz Centre for Environmental Research – UFZ, Department of Molecular Environmental Biotechnology, Permoserstraße 15, 04318 Leipzig, Germany, Tel.: +49 (0) 341 6025 2487, E-Mail: chang.ding@ufz.de

List of Supplementary Figures

Supplementary Figure S1. Yield of "*Ca.* Kuenenia stuttgartiensis" strain CSTR1 protein from different disruption methods. 3
Supplementary Figure S2. Effect of pH on nitrite-reducing activity in cell extracts of "*Ca.* Kuenenia stuttgartiensis" strain CSTR1. 4
Supplementary Figure S3. Effect of temperature on nitrite-reducing activity in cell extracts of "*Ca.* Kuenenia stuttgartiensis" strain CSTR1. 5
Supplementary Figure S4. Nitrogen flushing of headspace enhanced nitrite-reducing activity in cell extracts of "*Ca.* Kuenenia stuttgartiensis" strain CSTR1. 6
Supplementary Figure S5. Effect of phenazine methosulfate (PMS), phenazine ethosulfate (PES), and headspace volume on nitrite-reducing activity in cell extracts of "*Ca.* Kuenenia stuttgartiensis" strain CSTR1. 7
Supplementary Figure S6. Effect of ascorbic acid on nitrite reduction in cell extracts of "*Ca.* Kuenenia stuttgartiensis" strain CSTR1. 8
Supplementary Figure S7. Absorption profiles (colored lines) as well as distribution of nitrite-reducing activity (striped bars) "*Ca.* Kuenenia stuttgartiensis" strain CSTR1 after fractionation by size-exclusion chromatography. 9
Supplementary Figure S8. Distribution of proteins of "*Ca.* Kuenenia stuttgartiensis" strain CSTR1 (colored lines) that have the best match to the nitrite reducing activity profile (black dashed line) across the fractions of a size-exclusion chromatography run (0.1 mL per fraction between 12.5 and 13.4 mL elution volume). 10
Supplementary Figure S9. Absorption profiles (colored lines) and distribution of nitrite-reducing activity (striped bars) of "*Ca.* Kuenenia stuttgartiensis" strain CSTR1 after fractionation by anion exchange chromatography. 12
Supplementary Figure S10. Three fractions of "*Ca.* Kuenenia stuttgartiensis" strain CSTR1 obtained after ultracentrifugation at 130,000 × *g* for 3 hours. 13
Supplementary Figure S11. Recovery of nitrite-reducing activity of "*Ca.* Kuenenia stuttgartiensis" strain CSTR1 after ultracentrifugation at 130,000 × *g* for 3 hours. 14
Supplementary Figure S12. Correlation between protein distribution scores and the fraction in which the maximum amount of the protein was eluting in size exclusion chromatography (SEC)........ 15

Supplementary Figure S13. Recovery of nitrite-reducing activity of “*Ca*. Kuenenia stuttgartiensis” strain CSTR1 after ultrafiltration with 3 kDa and 100 kDa molecular filter... 16

List of Supplementary Tables

Supplementary Table S1. Protein distribution in fractions of “*Ca*. Kuenenia stuttgartiensis” strain CSTR1 cell extracts separated by ultracentrifugation. KUST: locus tag in “*Ca*. Kuenenia stuttgartiensis” KUST genome. 17
Supplementary Table S2. Protein distribution in fractions of prefractionated “*Ca*. Kuenenia stuttgartiensis” strain CSTR1 cell extracts separated by size exclusion chromatography (SEC). 17
Supplementary Table S3. Protein distribution in fractions of “*Ca*. Kuenenia stuttgartiensis” strain CSTR1 cell extracts separated by size exclusion chromatography. 17
Supplementary Table S4. Protein distribution in fractions of “*Ca*. Kuenenia stuttgartiensis” strain CSTR1 cell extracts separated by size exclusion chromatography. 17
Supplementary Table S5. Protein distribution in fractions of “*Ca*. Kuenenia stuttgartiensis” strain CSTR1 cell extracts separated by anion exchange chromatography (AEC). Fractions (0.2~0.4 mL each) were collected between 8.7 mL and 22.7 mL... 17
Supplementary Table S6. Proteins that matched the distribution of nitrite-reducing activity in the size exclusion chromatographic run as described in Figure 6. 17
Supplementary Table S7. Nitrite reductases detected using two nitrite reductase databases (a collection of 1.309 sequences from UniProt/Swiss-Prot and a compiled collection of NirS/NirK sequences by Pold *et al.*, 2024)... 17

Supplementary Figures

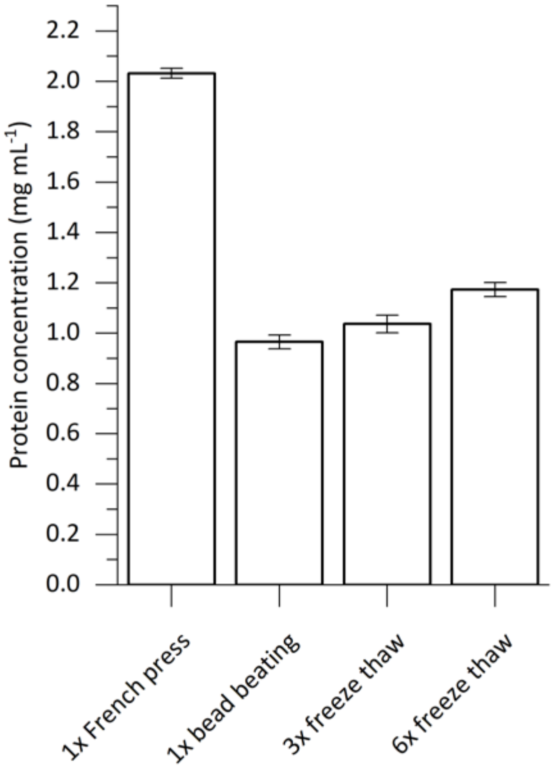

Supplementary Figure S1. Yield of “*Ca.* Kuenenia stuttgartiensis” strain CSTR1 protein from different disruption methods.

Cell density before disruption was 2.5×10^{10} cells mL^{-1}. French press: one passage of French press at a pressure of 137 MPa; bead beating: 4 m s^{-1} using 0.1 mm glass beads, 20 s per cycle, 3 cycles, ice bath in between; Freeze thaw: 3 or 6 cycles of 5 min ethanol bath at -80ºC and 1 min thawing at 40ºC with slight shaking. Protein concentrations of the supernatant after centrifugation at 16,100 × *g* for 10 min were measured with the BCA Protein Assay Kit (Pierce, Thermo Fisher Scientific). Data shows means of triplicate samples ± SD.

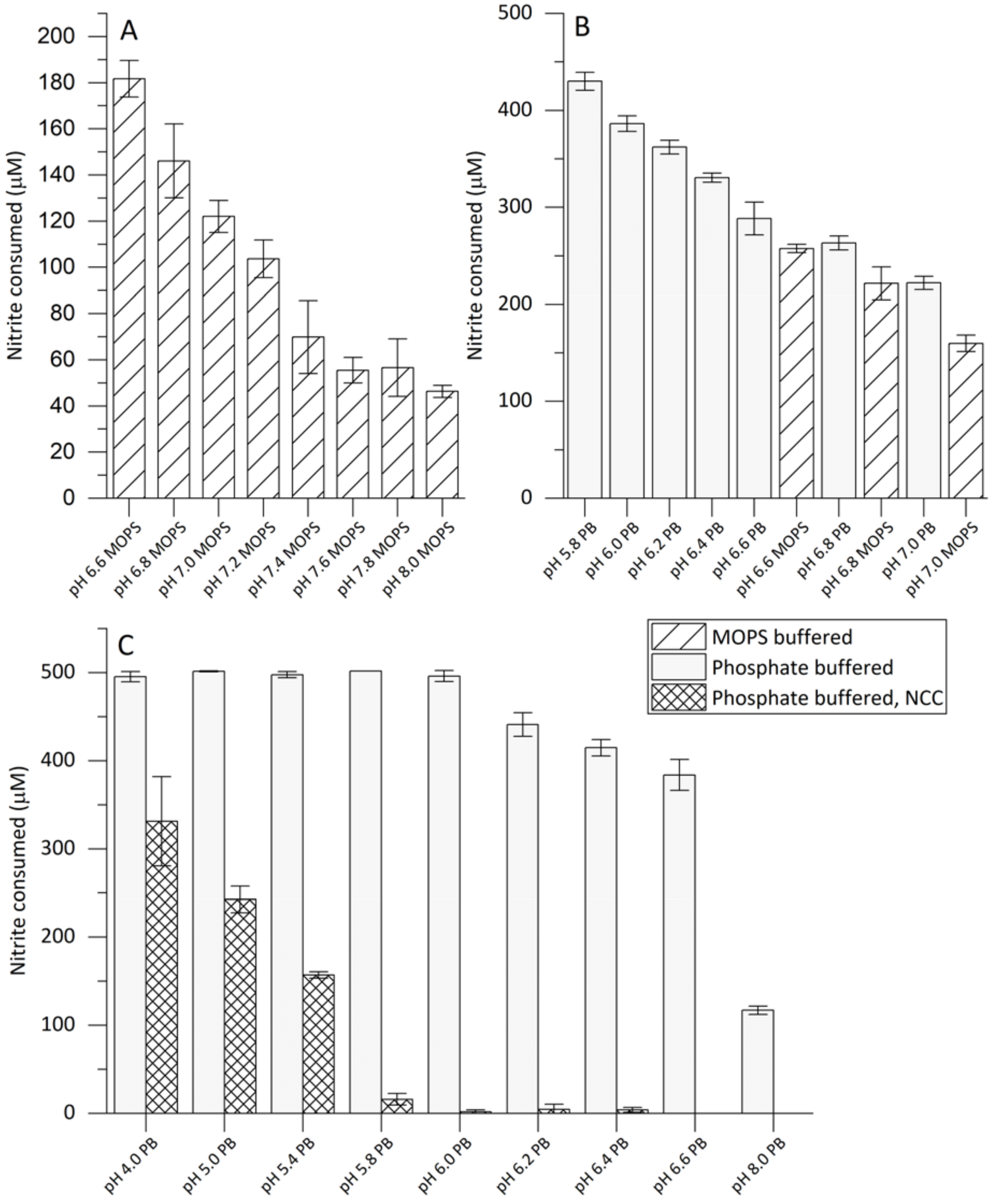

Supplementary Figure S2. Effect of pH on nitrite-reducing activity in cell extracts of "*Ca.* Kuenenia stuttgartiensis" strain CSTR1.

(A) pH range 6.6 to 8.0 with MOPS as the buffering agent, (B) pH range 5.8 to 7.0 with phosphate (PB) or MOPS as the buffering agent, (C) pH 4.0 to 8.0 with phosphate as the buffering agent. Activity assay contained 20 mM phosphate or MOPS buffer at various pH values, 0.5 mM ascorbic acid, 0.2 mM phenazine ethosulfate, 0.5 mM sodium nitrite, 1% v/v cell extracts from 100× concentrated cells (2.5×10^{8} cells mL^{-1}, protein concentration 2.0 mg mL^{-1}). Incubation time: 4 h for A and B, 2 h for C. NCC: no cell extract control. Data shows means of triplicate samples ± SD.

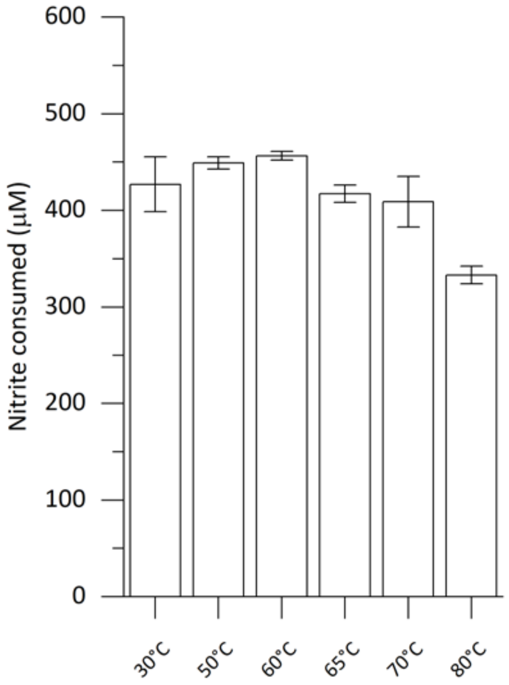

Supplementary Figure S3. Effect of temperature on nitrite-reducing activity in cell extracts of "*Ca.* Kuenenia stuttgartiensis" strain CSTR1.

The activity assay contained 20 mM phosphate buffer (pH 6.2), 0.5 mM ascorbic acid, 0.2 mM phenazine ethosulfate, 0.5 mM sodium nitrite, and 1% v/v cell extracts from 100× concentrated cells (2.5×10^8 cells mL^{-1}, protein concentration 2.0 mg mL^{-1}). Incubation time: 1 hour. Data shows means of triplicate samples ± SD.

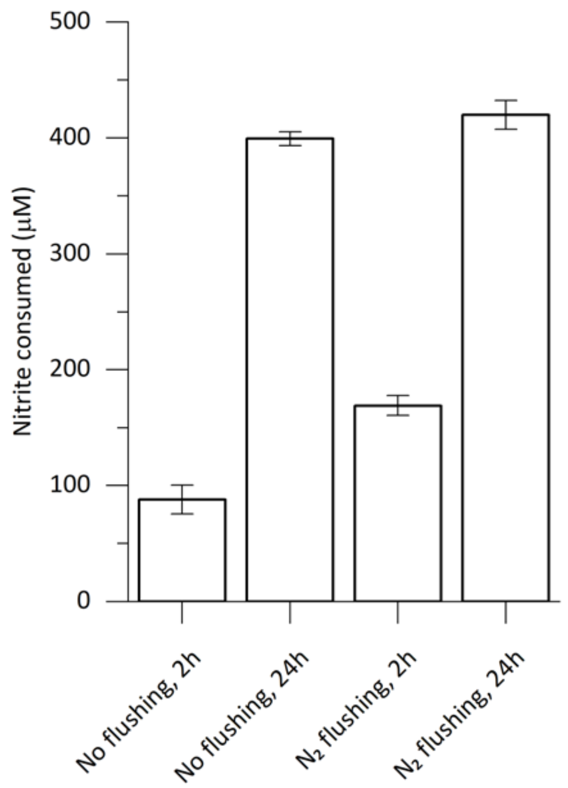

Supplementary Figure S4. Nitrogen flushing of headspace enhanced nitrite-reducing activity in cell extracts of "*Ca.* Kuenenia stuttgartiensis" strain CSTR1.

The activity assay contained 20 mM MOPS buffer at pH 7.2, 0.5 mM ascorbic acid, 1 mM phenazine methosulfate, 0.5 mM sodium nitrite, 1% v/v cell extracts from 100× concentrated cells (2.5×10^8 cells mL^{-1}, protein concentration 2.0 mg mL^{-1}). The no-flushing reactions were done in 5 mL reaction volumes using 10 mL vials. Reactions with nitrogen flushing were done in 30 mL reaction volume using 50 ml vials. Reactions were incubated for either 2 or 24 hours. Data shows means of triplicate samples ± SD.

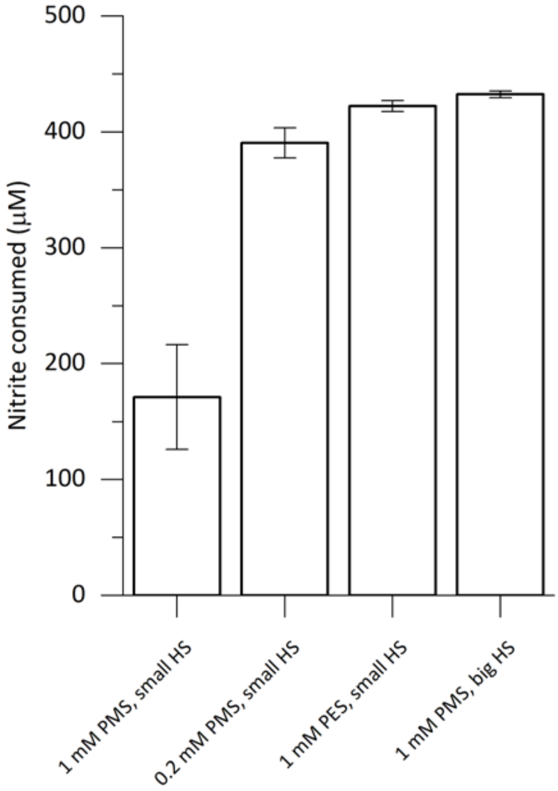

Supplementary Figure S5. Effect of phenazine methosulfate (PMS), phenazine ethosulfate (PES), and headspace volume on nitrite-reducing activity in cell extracts of “*Ca.* Kuenenia stuttgartiensis” strain CSTR1.

The activity assay contained 20 mM MOPS buffer at pH 7.2, 0.5 mM ascorbic acid, PMS or PES at concentrations indicated in the column label, 0.5 mM sodium nitrite, 1% v/v cell extracts from 100× concentrated cells (2.5×10^8 cells mL^{-1}, protein concentration 2.0 mg mL^{-1}). Reactions with small headspace were carried out in 5 mL reaction volume using 10 mL vials. Reactions with big headspace were carried out in 5 mL reaction volume using 200 mL bottles. Vials were incubated overnight. HS: headspace. Data shows means of triplicate samples ± SD.

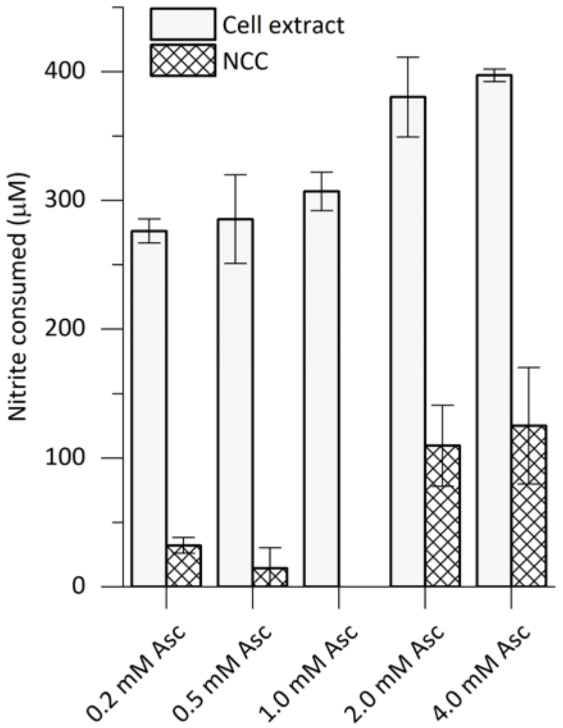

Supplementary Figure S6. Effect of ascorbic acid on nitrite reduction in cell extracts of "*Ca.* Kuenenia stuttgartiensis" strain CSTR1.

The activity assay contained 200 mM phosphate buffer (pH 6.2), various concentrations of ascorbic acid, 0.2 mM phenazine ethosulfate, 0.5 mM sodium nitrite, and 2% v/v cell extracts from 100× concentrated cells (2.5×10^{8} cells mL^{-1}, protein concentration 2.0 mg mL^{-1}). Incubation time: 1.5 hours. NCC is the "no-cell control", containing PBS instead of cells. Asc: ascorbic acid. Data shows means of triplicate samples ± SD.

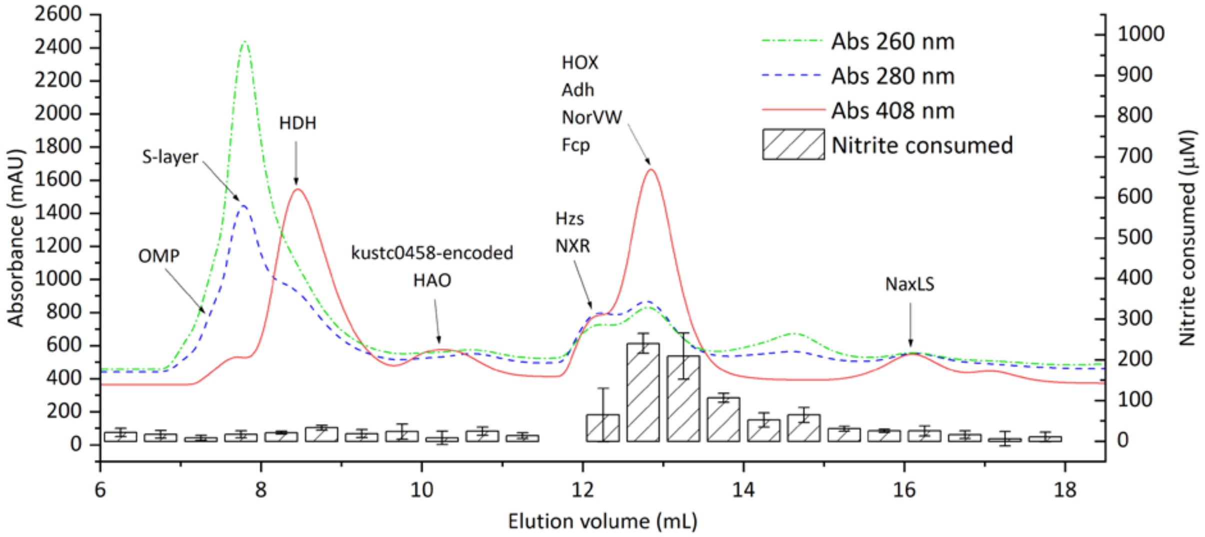

Supplementary Figure S7. Absorption profiles (colored lines) as well as distribution of nitrite-reducing activity (striped bars) "*Ca.* Kuenenia stuttgartiensis" strain CSTR1 after fractionation by size-exclusion chromatography.

Absorption at 280 nm indicates the presence of protein, whereas the absorption at 408 nm indicates the presence of heme. Volumes of 0.5 mL were taken for each fraction between 6 and 18 mL elution volume. Proteins of interests identified in the fractions by protein mass spectrometry are indicated on the absorption peaks. Protein amount injected: 200 µL cell extracts of from 100× concentrated cells (2.5×10^{8} cells mL^{-1}, protein concentration 2.0 mg mL^{-1}). The activity assay contained 200 mM phosphate buffer (pH 6.2), 1.0 mM ascorbic acid, 0.2 mM phenazine ethosulfate, 0.5 mM sodium nitrite, and 16.7% v/v eluted fraction. Incubation time: 4 hours. Data of nitrite reductase activity shows means of triplicate determination ± SD. The fraction 11.5–12 mL was lost.

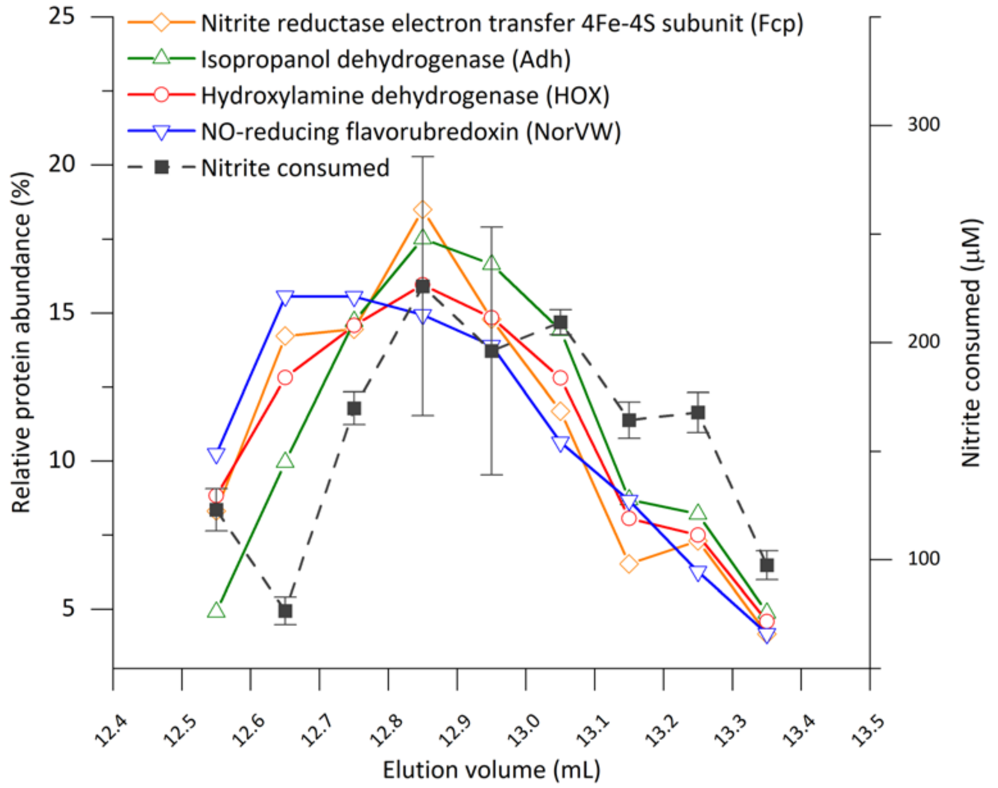

Supplementary Figure S8. Distribution of proteins of "*Ca.* Kuenenia stuttgartiensis" strain CSTR1 (colored lines) that have the best match to the nitrite reducing activity profile (black dashed line) across the fractions of a size-exclusion chromatography run (0.1 mL per fraction between 12.5 and 13.4 mL elution volume).

The activity assay contained 200 mM phosphate buffer (pH 6.2), 0.5 mM ascorbic acid, 0.2 mM phenazine ethosulfate, 0.5 mM sodium nitrite, and 16.7% v/v eluted fraction. Incubation time: 2.5 hours. The values for nitrite consumption show means of triplicate measurements of a fraction ± SD.

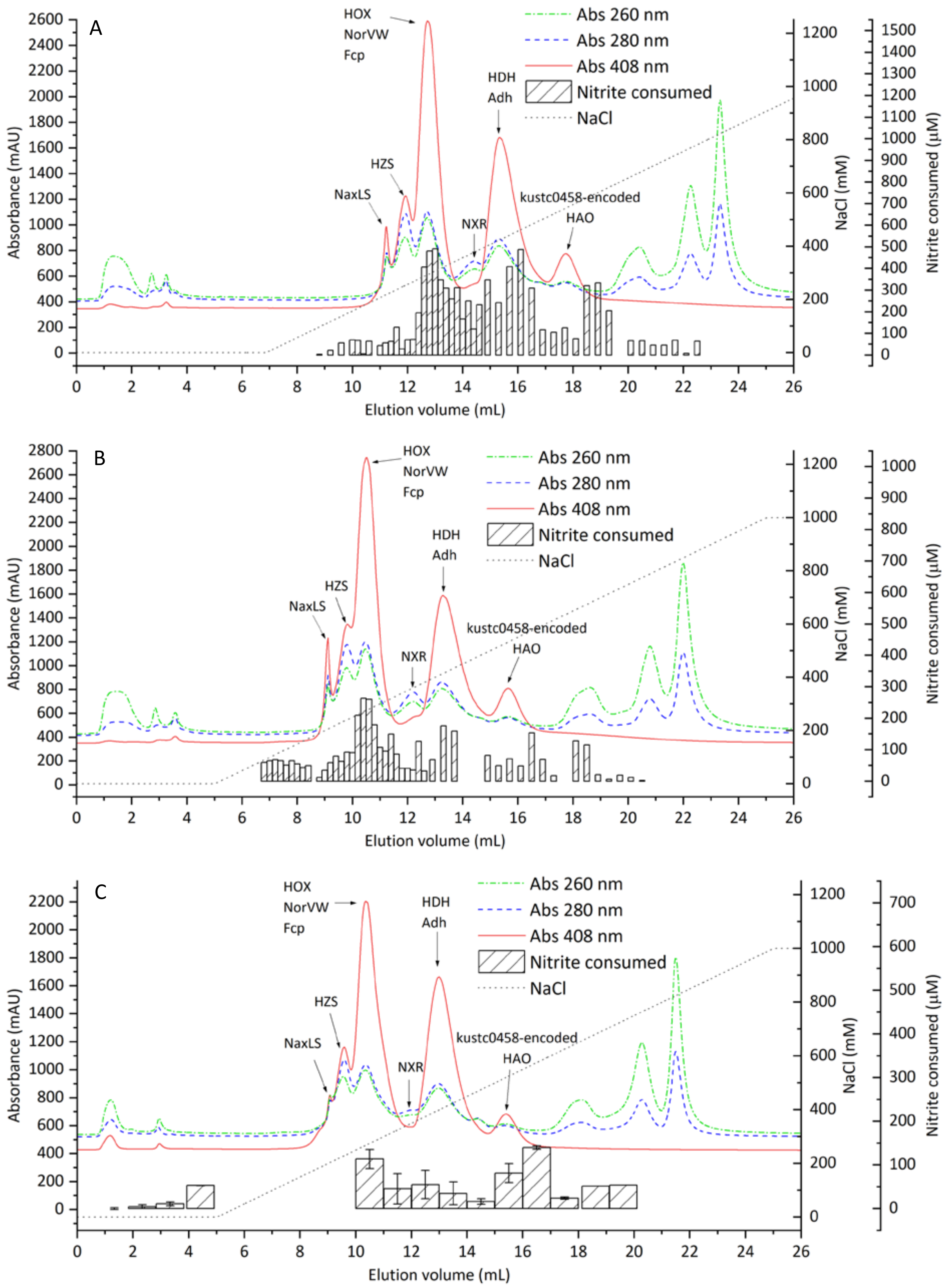
A
B
C
HOX
NorVW
Fcp
HZS
NaxLS
HDH
Adh
NXR
kustc0458-encoded
HAO
Abs 260 nm
Abs 280 nm
Abs 408 nm
Nitrite consumed
NaCl
Absorbance (mAU)
NaCl (mM)
Nitrite consumed (µM)
Elution volume (mL)

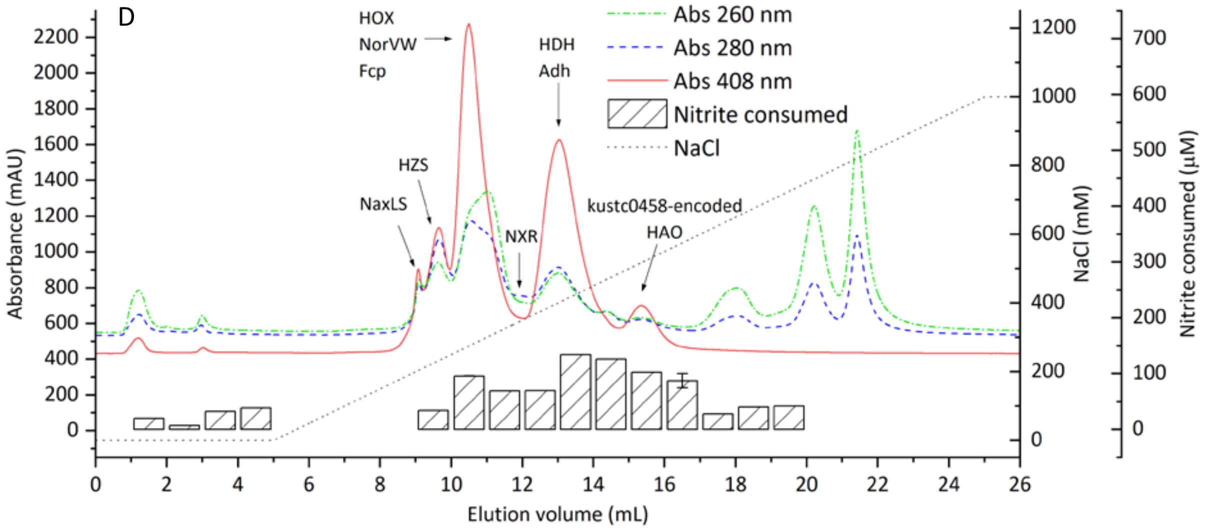

Supplementary Figure S9. Absorption profiles (colored lines) and distribution of nitrite-reducing activity (striped bars) of "*Ca.* Kuenenia stuttgartiensis" strain CSTR1 after fractionation by anion exchange chromatography.

Inconsistent and low activities were observed. The biological material injected was 500 µL cell extracts from 100× concentrated cells (2.5×10^8 cells mL^{-1}, protein concentration 2.0 mg mL^{-1}). The panels show different incubation times for the activity test: A) 18 hours, B) 4 hours, C) 3 hours, D) 6 hours. (A) has associated proteomics data in Supplementary Table S5.

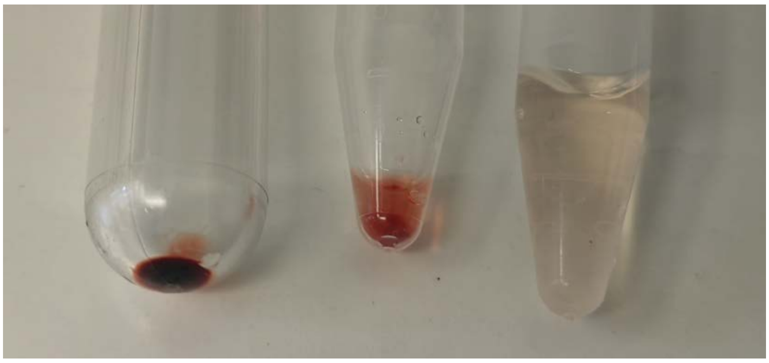

Supplementary Figure S10. Three fractions of "*Ca.* Kuenenia stuttgartiensis" strain CSTR1 obtained after ultracentrifugation at 130,000 × *g* for 3 hours.

From left to right: pelleted membrane, intermediate fraction that was immediately above the pellet, and cytosolic fraction.

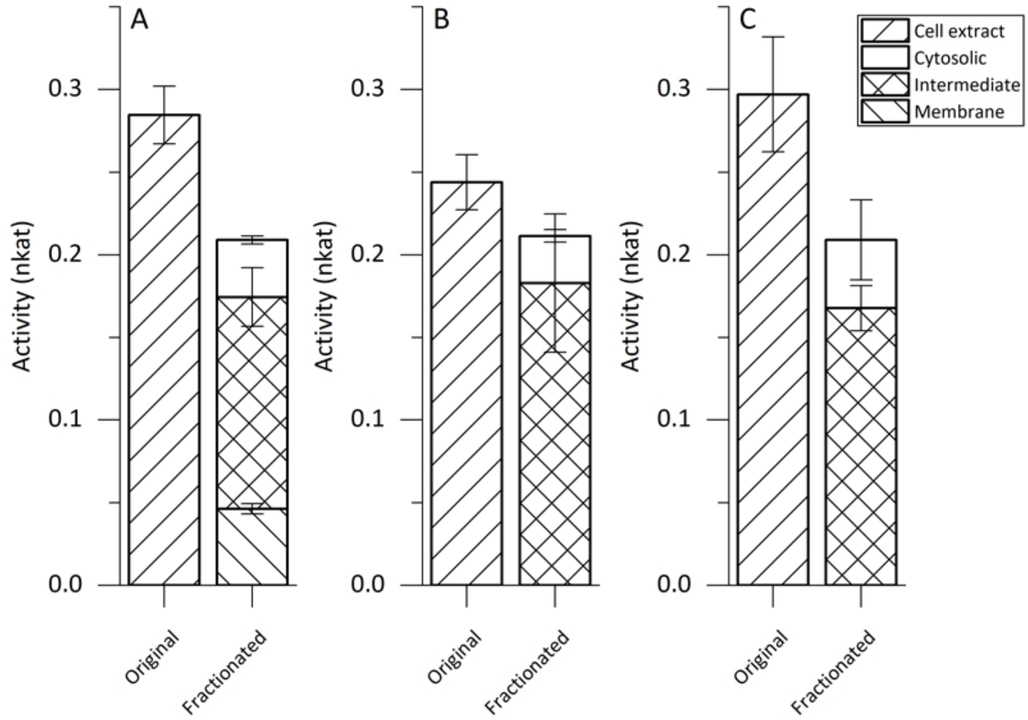

Supplementary Figure S11. Recovery of nitrite-reducing activity of "*Ca.* Kuenenia stuttgartiensis" strain CSTR1 after ultracentrifugation at 130,000 × *g* for 3 hours.

Activity shown was corresponding to 1 mg starting protein amount. The panels (A), (B), and (C) show three independent experiments. Orbitrap results (Supplementary Table S1) are associated with (A). Associated with the data shown in (C) is the ultrafiltration data shown in Supplementary Figure S13. Pelleted membrane fraction was resuspended in 1× PBS containing 1% w/v n-dodecyl-β-D-maltoside (DDM). The activity assay contained 200 mM phosphate buffer (pH 6.2), 0.5 mM ascorbic acid, 0.2 mM phenazine ethosulfate, 0.5 mM sodium nitrite, 2% v/v cell extract or fraction. Incubation time: 2 hours. Data shows means of triplicate samples ± SD.

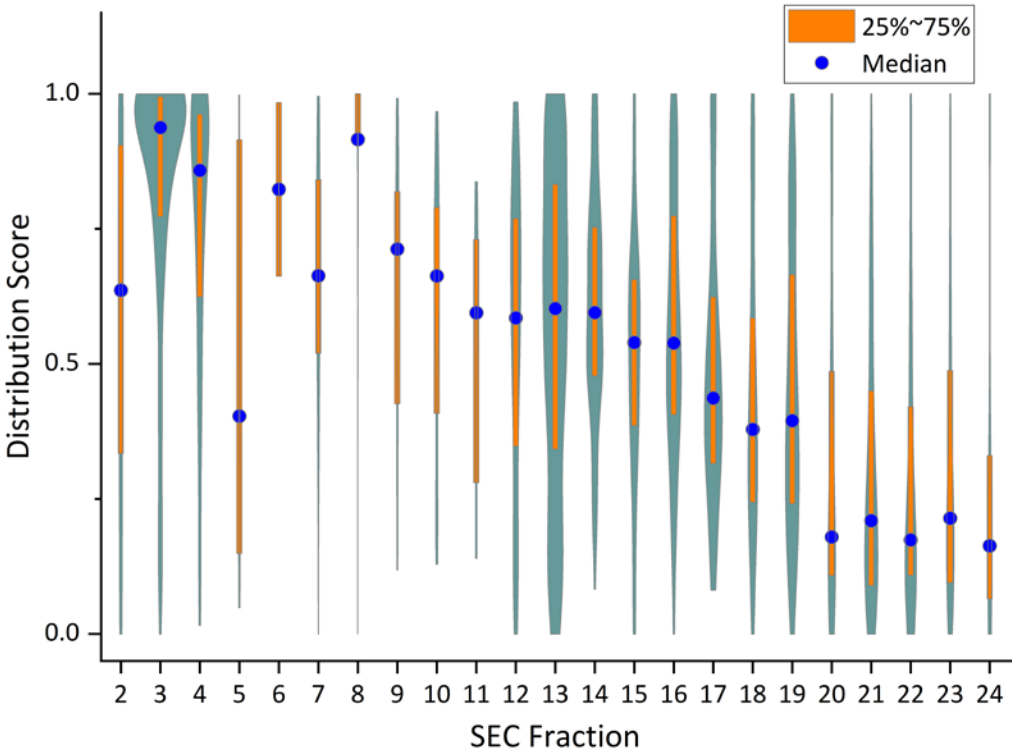

Supplementary Figure S12. Correlation between protein distribution scores and the fraction in which the maximum amount of the protein was eluting in size exclusion chromatography (SEC).

Assignment of proteins to SEC fractions was done according to the SEC data in Supplementary Table S3. Protein distribution score = percentage in membrane fraction × 1.0 + percentage in intermediate fraction × 0.5 (based on proteomics data in Supplementary Table S1). A score closer to 1 indicates higher abundance in the membrane fraction.

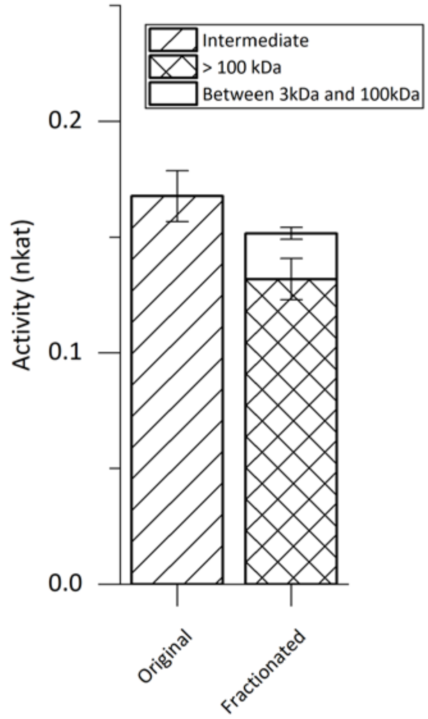

Supplementary Figure S13. Recovery of nitrite-reducing activity of "*Ca.* Kuenenia stuttgartiensis" strain CSTR1 after ultrafiltration with 3 kDa and 100 kDa molecular filter.

Activity shown was corresponding to 1 mg starting protein amount. Starting material was the intermediate fraction obtained after ultracentrifuging the cell extract at 130,000 × *g* for 3 hours (Supplementary Figure S11C). The activity assay contained 200 mM phosphate buffer (pH 6.2), 0.5 mM ascorbic acid, 0.2 mM phenazine ethosulfate, 0.5 mM sodium nitrite, 2% v/v fraction. Incubation time: 2 hours.

Supplementary Tables

All supplementary tables are contained in the excel file: Supplementary_Tables_S1_to_S7.xlsx

Supplementary Table S1. Protein distribution in fractions of "*Ca.* Kuenenia stuttgartiensis" strain CSTR1 cell extracts separated by ultracentrifugation. KUST: locus tag in "*Ca.* Kuenenia stuttgartiensis" KUST genome.

Protein distribution score was calculated as follows: percentage in membrane fraction × 1.0 + percentage in intermediate fraction × 0.5. SEC fraction: fraction in the SEC run (Supplementary Table S3) where the protein showed the highest abundance.

Supplementary Table S2. Protein distribution in fractions of prefractionated "*Ca.* Kuenenia stuttgartiensis" strain CSTR1 cell extracts separated by size exclusion chromatography (SEC).

Starting material for SEC was the fraction with size >100 kDa after ultracentrifugation (taking the intermediate fraction) and ultrafiltration (Supplementary Figure S13). Fractions (0.5 mL each) were collected between 6 mL and 21 mL. Duplicate proteomics measurements were done with fractions F12-F17 (11.5–14.5 mL) where nitrite-reducing activity appeared. Corresponding SEC results and activities of fractions were shown in Figure 6. KUST: locus tag in "*Ca.* Kuenenia stuttgartiensis" KUST genome.

Supplementary Table S3. Protein distribution in fractions of "*Ca.* Kuenenia stuttgartiensis" strain CSTR1 cell extracts separated by size exclusion chromatography.

Fractions (0.5 mL each) were collected between 6 mL and 18 mL. KUST: locus tag in "*Ca.* Kuenenia stuttgartiensis" KUST genome.

Supplementary Table S4. Protein distribution in fractions of "*Ca.* Kuenenia stuttgartiensis" strain CSTR1 cell extracts separated by size exclusion chromatography.

Fractions (0.1 mL each) were collected between 11.5 mL and 12.9 mL. KUST: locus tag in "*Ca.* Kuenenia stuttgartiensis" KUST genome.

Supplementary Table S5. Protein distribution in fractions of "*Ca.* Kuenenia stuttgartiensis" strain CSTR1 cell extracts separated by anion exchange chromatography (AEC). Fractions (0.2~0.4 mL each) were collected between 8.7 mL and 22.7 mL.

Data from F37 and F48 were discarded due to problems during mass acquisition. Corresponding AEC curve and activities of fractions were shown in Supplementary Figure S9A. Distribution of selected proteins were shown in Figure 5. KUST: locus tag in "*Ca.* Kuenenia stuttgartiensis" KUST genome.

Supplementary Table S6. Proteins that matched the distribution of nitrite-reducing activity in the size exclusion chromatographic run as described in Figure 6.

The protein distribution in fractions F12-F17 was considered a match to the activity distribution, if the protein abundances follow F14 >F15>F13>/≈F16>F12>/≈F17. KUST: locus tag in "*Ca.* Kuenenia stuttgartiensis" KUST genome. Complete distribution of proteins can be found in Supplementary Table S2. Proteins in bold were those mentioned in discussion.

Supplementary Table S7. Nitrite reductases detected using two nitrite reductase databases (a collection of 1.309 sequences from UniProt/Swiss-Prot and a compiled collection of NirS/NirK sequences by Pold *et al.*, 2024)
